# NIH4215: A mutation-prone thiamine auxotrophic clinical *Aspergillus fumigatus* isolate

**DOI:** 10.3389/ffunb.2022.908343

**Published:** 2022-07-25

**Authors:** Roberta Peres da Silva, Matthias Brock

**Affiliations:** University of Nottingham, School of Life Sciences, University Park, Nottingham, United Kingdom

**Keywords:** *nmt1*, thiamine biosynthesis, bioluminescent reporter, *akuB*, spontaneous mutations, DHN-melanin, *Galleria mellonella*

## Abstract

*Aspergillus fumigatus* is the main cause of life-threatening invasive aspergillosis. Despite the availability of various antifungals, therapy remains challenging and requires further studies. Accordingly, the clinical *A. fumigatus* isolate NIH4215 deriving from a fatal case of human pulmonary aspergillosis has frequently been used in drug efficacy studies. Unexpectedly, our initial attempts to generate a bioluminescent reporter of strain NIH4215 for *in vivo* drug efficacy studies failed, as NIH4215 was unable to grow on defined minimal medium. Subsequent analyses discovered a previously undescribed thiamine auxotrophy of strain NIH4215 and transformation with thiamine biosynthesis genes from *A. fumigatus* strain Af293 identified the *nmt1* gene as cause of the thiamine auxotrophy. Sequencing of the defective *nmt1* gene revealed the loss of a cysteine codon within an essential iron-binding motif. Subsequently, the wild-type *nmt1* gene was successfully used to generate a bioluminescent reporter strain in NIH4215 by simultaneously deleting the *akuB* locus. The resulting bioluminescent Δ*akuB* strains showed a high frequency of homologous integration as confirmed by generation of *pyrG* and *niaD* deletion mutants. When tested in a *Galleria mellonella* infection model, neither thiamine auxotrophy nor the deletion of the *akuB* locus had a significant effect on virulence. However, besides thiamine auxotrophy, sectors with altered morphology and albino mutants frequently arose on colony edges of strain NIH4215 and its derivatives, and stable albino mutants were successfully isolated. A proposed increased mutation rate of NIH4215 was confirmed by screening for spontaneous occurrence of fluoorotic acid resistant mutants. Independent mutations in the *pyrG* and *pyrE* gene were identified in the fluoroorotic acid resistant NIH4215 isolates and the frequency of mutation was by at least one order of magnitude higher than that observed for the clinical *A. fumigatus* isolate CBS144.89. In summary, despite its virulence in animal models, strain NIH4215 is a thiamine auxotroph and prone to accumulate mutations. Our results suggest that thiamine biosynthesis is dispensable for host infection and mutation-prone strains such as NIH4215 could potentially facilitate the evolution of azole resistant strains as increasingly observed in the environment.

## Introduction

*Aspergillus fumigatus* is the main cause of life-threatening invasive bronchopulmonary aspergillosis, which mainly affects patients suffering from haematological malignancies or recovering from solid organ transplantation (Pagano et al., 2011). However, it also causes severe complications in critically ill patients suffering from influenza or SARS-CoV2 infections (Montrucchio et al., 2021; Shi et al., 2022). Despite extensive research on immune responses contributing to fungal elimination, the investigation of fungal virulence determinants, and studies on antifungal drug efficacy, mortality rates from invasive aspergillosis remain high with 50 - 90% of patients succumbing to infection (Kousha et al., 2011). Therefore, further studies are needed to analyse the pathobiology of *A. fumigatus* and to test the efficacy of antifungal drugs under different immunosuppressive regimens.

Bioluminescence imaging in combination with other *in vivo* imaging techniques has opened new avenues to study fungal disease progression in temporal and spatial resolution in murine infection models (Jacobsen et al., 2014; Poelmans et al., 2018; Persyn et al., 2019; Vanherp et al., 2019; Vanherp et al., 2020; Seldeslachts et al., 2021). Currently, one of the most suitable reporters for bioluminescence imaging of fungal diseases relies on codon optimised firefly luciferases. Such optimised luciferases have been successfully used to study disease progression and antifungal therapy in filamentous fungi such as *A. fumigatus* (Galiger et al., 2013; Poelmans et al., 2018; Seldeslachts et al., 2021; Resendiz-Sharpe et al., 2022) and *Aspergillus terreus* (Slesiona et al., 2012). In addition several other studies focussed on pathogenic yeasts such as biofilm attachment, localised and disseminated organ infection and persistence of *Candida albicans* in the gall bladder under antifungal therapy (Jacobsen et al., 2014; Vande Velde et al., 2018), *in vivo* biofilm formation and urinary tract persistence of *Candida glabrata* (Persyn et al., 2019; Schrevens and Sanglard, 2021) and dissemination of *Cryptoccocus neoformans* to the brain (Vanherp et al., 2019).

To investigate the contribution of individual virulence determinants in pathogenesis of *A. fumigatus*, deletion mutants are frequently generated and tested for their virulence potential. However, this approach is hampered by a limited availability of transformation markers and, moreover, a preference of ectopic integration of gene deletion constructs due to a highly active non-homologous end-joining (NHEJ) repair mechanism in *Aspergillus* wild-type strains (Takahashi et al., 2006; Chang, 2008; Zhang et al., 2011; Zhang et al., 2016). To overcome the latter problem, it has been shown that deletion of either one of the homologues of human KU^70^ and KU^80^ encoding genes, which are called *akuA* and *akuB* in *A. fumigatus*, significantly improves the number of transformants with homologous integration (Da Silva Ferreira et al., 2006; Krappmann et al., 2006). In addition, these strains display a virulence potential in murine infection models comparable to that of parental strains (Da Silva Ferreira et al., 2006).

To combine the benefits of bioluminescence imaging with the increased rate of homologous recombination, we aimed in the construction of an *A. fumigatus* strain in which a codon-optimised red-shifted luciferase reporter replaces the *akuB* coding region in NIH4215 (ATCC MYA-1163) as previously performed on the clinical isolate CBS144.89 (Resendiz-Sharpe et al., 2022).

NIH4215 derived from a patient succumbing to a fatal case of human invasive pulmonary aspergillosis (Petraitis et al., 2003) and was of special interest as the strain had been applied to several studies investigating the efficacy of antifungal treatment in various *in vitro* and *in vivo* infection models (Petraitis et al., 2003; Such et al., 2013; Box et al., 2016; Hope et al., 2017). Unexpectedly, while strain NIH4215 showed normal growth on a range of complex media such as Sabouraud dextrose medium, cell-culture media or Potato dextrose broth (Box et al., 2016; Hope et al., 2017), we observed that the strain failed to grow on a defined synthetic *Aspergillus* minimal medium. Therefore, we investigated the underlying cause of the growth deficiency of NIH4215 and identified the mutation causing the growth defect. A wild-type version of the affected gene was then used to generate a bioluminescent reporter strain by replacing the *akuB* locus by a red-shifted codon-optimised luciferase. Subsequent analyses of strain NIH4215 and the derived reporter strains revealed that this clinical *A. fumigatus* isolate is highly prone to accumulate stable mutations in its genome.

## Methods

### Strains, media and general cultivation conditions

Wild-type strains used in this study were NIH4215 from the American Type Culture collection (ATCC; strain also found under the name MYA-1163) and an independent sample was kindly provided by T. Lehrnbecher, Goethe-University Frankfurt/Main, Germany). The genome sequenced *A. fumigatus* strain Af293 (also known as ATCC strain MYA-4609) and strain CBS144.89 (Westerdijk Fungal Biodiversity Institute; also known as Dal strain and parental strain of CEA10 (D’enfert et al., 1996) which is the source of the second reference genome “A1163”) were used as controls in individual experiments. Reactivation of strains from cryostocks was performed on agar slants containing malt extract medium (Sigma-Aldrich) that was solidified by the addition of 2% agar. *Aspergillus* minimal medium (AMM) was prepared by using per litre of medium: 0.52 g KC1, 0.52 g MgSO_4_ * 7H_2_0, 1.52 g KH_2_PO_4_, 9.6 g glucose monohydrate, and 1 ml of Hutner’s trace element solution (http://www.fgsc.net/methods/anidmed.html). As standard nitrogen source 1.46 g/l of l-glutamine (10 mM) was added. The pH of the medium was adjusted to 6.5 using 10 M NaOH. For solid media, 20 g/l of agar was added and all media were sterilised by autoclaving. If not indicated otherwise, all growth experiments were performed at 37°C and liquid cultures were agitated on a rotary shaker at 150 rpm. For inoculation of media with a defined concentration of conidia, conidia were harvested from agar slants by overlaying the colonies with 10 ml of phosphate-buffered saline (PBS) containing 0.01% Tween 80 and conidia were brought into suspension using a sterile cotton swap. Suspensions were filtered over 40 µm cell strainers (EASYstrainer, Greiner bio-one), centrifuged for 10 min at 4000 × *g* and resuspended in 4 ml of PBS. Conidia were counted using a Neubauer counting chamber and dilutions for spotting experiments were prepared to obtain the desired number of conidia in a total volume of 4 µl. The indicated amount of conidia was spotted on plates and incubation was started once the liquid had been completely absorbed by the underlying solid medium. Cryostocks of selected strains were prepared in Microbank cryovials (Pro-Lab Diagnostics) and stored at -80°C. A single bead was removed for strain reactivation.

### Vitamin supplementation

Initial testing of vitamin requirements of strain NIH4215 was performed by using a vitamin stock solution as used for the supplementation of *Candida albicans* minimal medium (Otzen et al., 2013) with a composition per 100 mL in 20% ethanol of 2 mg biotin, 20 mg thiamine/HCl and 20 mg pyridoxine/HCl. This vitamin stock was used at a final concentration of 0.03%. Alternatively, individual vitamins were added in the concentrations as described for the vitamin stock or as described in individual experiments. When analysing the concentration of thiamine required to complement the growth defect of strain NIH4215, a starting concentration of 25 µg/ml was used followed by two-fold serial dilutions. Resistance against pyrithiamine was tested by the addition of pyrithiamine hydrobromide (Sigma) in a final concentration of 0.1 µg/ml.

### Imaging of light emission from bioluminescent reporter strains

To analyse strains for the functional expression of a codon-optimised red-shifted luciferase, molten growth media were supplemented with d-luciferin potassium salt (Perkin Elmer) to give a final concentration of 0.3 mM. At indicated time points, plates were removed from the incubator and placed in the imaging chamber of a Bio-Rad gel-doc system equipped with a XRS+ camera. All filters were removed and the first picture was recorded under illumination to visualise the colonies present on the plate. A second picture was taken in the dark by opening the camera lens and using a picture acquisition time of 60 seconds. The pixel binning was kept at the highest resolution (254 dpi, lowest sensitivity) to keep the picture size identical to the illuminated picture. Pictures were exported to TIFF format.

### Growth and resistance analysis of Δ*pyrG* and Δ*niaD* strains

For *pyrG* negative strains media were supplemented with 1.12 g/l of uracil (10 mM) prior to autoclaving. Uridine was added to hot media from a sterile filtered 1 M stock solution to give a final concentration of 10 mM. To test for the resistance against 5-fluoroorotic acid (FOA), uracil containing *Aspergillus* minimal medium with glutamine as nitrogen source was buffered with 20 mM (final concentration) HEPES-buffer (pH 7.0) and up to 4 mg/ml of UV-sterilised FOA was added. The medium was briefly boiled in a microwave to ensure complete dissolving of FOA before pouring plates. To test the utilisation of various nitrogen sources by Δ*niaD* strains, *Aspergillus* minimal media containing 50 mM glucose were supplemented with a final concentration of either 10 mM glutamine, 10 mM urea, 20 mM acetamide or 70 mM nitrate. To test for the increased chlorate resistance of Δ*niaD* strains, minimal medium with 20 mM acetamide as nitrogen source was supplemented with 120 mM potassium chlorate. Resistance against 5-fluorouridine was tested by preparing glucose and glutamine containing media supplemented with 12.5 µg/ml of thiamine and 10 mM uracil and with or without the addition of 2 mM 5-fluorouridine.

### General molecular biology methods

For transformation of *A. fumigatus* strains, a PEG-mediated protoplast transformation protocol was followed as previously described (Brock et al., 2008) with some minor modifications. These modifications included a 60 min pre-incubation of harvested mycelium at 28°C in 90 mM citrate-phosphate buffer pH 7.3 containing 10 mM dithiothreitol. In addition, the previously described cell-wall degrading enzymes were replaced by a mixture of 0.1 g lysing enzymes from *Trichoderma harzianum* (Sigma) and 1.3 g VinoTaste Pro (Novozymes) in 20 ml of osmotic medium (0.6 M KCl, 10 mM potassium phosphate pH 5.8). For transformation of *Escherichia coli*, DH5α cells were used that were made chemically competent by using the Mix & Go! *E. coli* Transformation Kit (Zymo Research). Restriction digests of DNA were performed using FastDigest restriction enzymes (Thermo Scientific). Plasmid DNA was isolated from *E. coli* using the NucleoSpin plasmid isolation kit (Machery-Nagel) as described in the manufacturer’s protocol. DNA fragments were eluted from agarose gels using the Zymoclean DNA recovery kit (Zymo Research). For amplification of DNA fragments used in the generation of reporter and deletion construct the Phusion proofreading polymerase was used that was replaced by the Phire polymerase in screening approaches (both Thermo Scientific). PCR reactions were performed in a SpeedCycler^2^ (Analytic Jena). Oligonucleotides used in this study were synthesised by Eurogentec (Kaneka Eurogentec S.A., Belgium) and are listed in **Table 1**.

**Table 1:** Oligonucleotides used in this study.

No.	Sequence (5’ – 3’)	Application
**1**	CAGCTGAAGCGTGTAGTTTGG	Amplification of *A. fumigatus thiE*
**2**	CTTGTTGGATGGATGCTTGAG	Amplification of *A. fumigatus thiE*
**3**	GCGGCCGCATTTGTATGTCTCGACAGGTC	Amplification of *A. fumigatus nmt1*
**4**	GCGGCCGCGAACCGCAATGATAGGTTG	Amplification of *A. fumigatus nmt1*
**5**	GTCCTGTATCCTCATTATCTGG	Amplification of *A. fumigatus thi4*
**6**	CGCTGAAGGAGTTTGAGTGG	Amplification of *A. fumigatus thi4*
**7**	GTACTACGAAGGCTGGCTGG	Amplification of *A. fumigatus tenA*
**8**	CTGTCTGTTCAGTTGTAGATCAC	Amplification of *A. fumigatus tenA*
**9**	GACGACCCTGAGGCGGCCGCGGGCTG	Cloning P*gpdA*:l*uc*__redTS_ into Δ*akuB*_pUC19 plasmid
**10**	ATACAAATGCGTGGTAGCTCGTTGTCGACG	Cloning P*gpdA*:l*uc*__redTS_ into Δ*akuB*_pUC19 plasmid
**11**	GAGCTACCACGCATTTGTATGTCTCGACAGGTC	Cloning Af293 *nmt1* into Δ*akuB*_pUC19 plasmid
**12**	CTTTAACTTTGGGCGGCCGCGATGCATACAAGGATCACTTCC	Cloning Af293 *nmt1* into Δ*akuB*_pUC19 plasmid
**13**	GACGACAAGCCCGGCGCC	Cloning control for Δ*akuB*:*luc*__redTS__*nmt1*_pUC19
**14**	GGATGACTCCAACCTCTGTC	Cloning control for Δ*akuB*:*luc*__redTS__*nmt1*_pUC19
**15**	CCATTTAGAGGAGCGACTTGC	*akuB* deletion control
**16**	CGACGAGACCGGGTAAGTTC	*akuB* deletion control
**17**	GCAAGGTTGACATGGGCTTC	*akuB* deletion control; *nmt1* sequencing
**18**	GCGGCCGCCCAAAGTTAAAGGGCGCAAGC	*akuB* probe for Southern blot
**19**	CTCTAGAGGATCCCCGGGCTATCACTTTGCCCAGTC	*akuB* probe for Southern blot
**20**	AATTCGAGCTCGGTACCCGGGAGTTACCAGTGATTGACC	Amplification upstream region *pyrG;* probe *pyrG* deletion
**21**	CCAAGAGCGGCCGCCGTGGTATTGCTTCTG	Amplification upstream region *pyrG;* probe *pyrG* deletion
**22**	CACGGCGGCCGCTCTTGGAGCAAAAGTGTAGTG	Amplification downstream region *pyrG*
**23**	CGACTCTAGAGGATCCCCGGGCACGCATTCCCGAGTGCAC	Amplification downstream region *pyrG*
**24**	GATTTGGTTGGGTCACCCTC	Control upstream and downstream regions of *pyrG* into pUC19
**25**	AATTCGAGCTCGGTACCCGGGTTGCTCTCTGCACCAAG	Amplification upstream region of *niaD*, probe *niaD* deletion
**26**	CACAACGCGGCCGCCATTGCTCAGAGTACTACAG	Amplification upstream region of *niaD* probe *niaD* deletion
**27**	CAATGGCGCGGCCGCGTTGTGTAGAATGGCTGCGAG	Amplification downstream region of *niaD*
**28**	CGACTCTAGAGGATCCCCGGGTCTCATTCGCGATTCAACTG	Amplification downstream region of *niaD*
**29**	CTTCTGTCTGGCCTATCCTTG	Control upstream and downstream regions of *niaD* into pUC19
**30**	TCCTCCAGCTGCCATCTAC	Cloning control of *ptrA* cassette in Δ*niaD*Af_pUC19
**31**	GCAATTGATTTGGTTGGGTCAC	Amplification *pyrG* from FOA mutants
**32**	CCTCATTGCTCGGCTACTTC	Forward *pyrG* sequencing primer
**33**	GACAGGCACAATGTATGCAGC	Reverse *pyrG* sequencing primer
**34**	GGCCAATACGGACGAGCTC	Amplification *pyrG* from FOA mutants
**35**	CTCTGCATTCGGTGATCTAA	Amplification *pyrE* from FOA mutants
**36**	CCTCATAACACCTCAGATCC	Forward *pyrE* sequencing primer
**37**	CTGTAAACTTCTGTTCATCAAGC	Amplification *pyrE* from FOA mutants

### Isolation of genomic DNA for transformant screening and for Southern blot analyses

To screen genomic DNA (gDNA) from Δ*akuB* transformants for the homologous integration of the deletion construct, a “chelex” DNA extraction method was applied. In brief: Individual wells of a 96-well microplate containing 200 µl of *Aspergillus* complete medium (http://www.fgsc.net/methods/anidmed.html) were inoculated with conidia from individual colonies using a 10 µl inoculation loop. The plate was sealed with a breathable film (AeraSeal, Excel Scientific) and incubated at 28°C for 16 h. A 5% Chelex 100 suspension (sodium form, Sigma) was prepared in water and 100 µl were transferred into 0.5 ml skirted micro tubes with screw-caps (Sarstedt) filled with 5 - 10 beads of lysing matrix D (MP Biomedicals). The mycelium was picked from the wells with a sterile toothpick (about 0.2 mm^3^), excess medium was removed with tissue paper and mycelium was transferred to the tubes. The mycelium was disrupted by two cycles of 1 min bead beating in a FastPrep-24 machine (MP Biomedicals) at 6000 rpm. Subsequently, samples were incubated for 5 min at 99°C, centrifuged at maximum speed in a microcentrifuge and 1 µl of the supernatant was used as template in PCR screening approaches. For Southern blot analyses gDNA was isolated by a protocol virtually as previously described (Dellaporta et al., 1983) and dried DNA was solved in 60 µl of 5 mM Tris-buffer pH 8.5. The quality of isolated DNA was checked by loading a 2 µl aliquot on a 0.8% agarose gel.

### Genetic complementation of thiamine auxotrophy of strain NIH4215

Candidate genes of *A. fumigatus* thiamine biosynthesis were identified by BLASTP analyses (Altschul et al., 1990) against the Af293 genome using *Saccharomyces cerevisiae* thiamine biosynthesis genes as templates. Results were used to confirm the coding and flanking DNA regions at FungiDB (https://fungidb.org/fungidb/app). Oligonucleotides were deduced to amplify fragments with about 1 kb upstream and 0.3 kb downstream regions of the respective thiamine biosynthesis genes from gDNA of strain Af293. The *thiE* gene (accession: XP_755230; locus tag AFUA2G08970) was amplified with oligonucleotides 1 and 2, the *nmt1* gene (accession: XP_748095; locus tag AFUA_5G02740) with oligonucleotides 3 and 4, the *thi4* gene (accession: XP_750752; locus tag AFUA_6G08360) with oligonucleotides 5 and 6 and the *tenA* gene (accession: XP_755470; locus tag AFUA_2G10740) with oligonucleotides 7 and 8. The PCR products were gel-purified and directly used for protoplast transformation of strain NIH4215. Thiamine-free glucose minimal medium containing 10 mM glutamine as nitrogen source (GG10) and 1.2 M sorbitol as osmotic stabiliser was used for regeneration of transformants. A gel-purified *nmt1* PCR product from strain NIH4215 was sequenced by Eurofins using oligonucleotides 3, 4 and 17.

### Generation of bioluminescent Δ*aku*B deletion mutants

To generate bioluminescent reporter strains by simultaneously deleting the *akuB* locus from strain NIH4215, we constructed the plasmid Δ*akuB*::*luc_OPT_*__red__*nmt1*_pUC19. The reporter containing the codon-optimised *luc_OPT_*__red_ gene (accession number: MT554554) under control of the promoter and terminator of the glyceraldehyde-3-phosphate dehydrogenase (*gpdA*) gene from *Aspergillus nidulans* was amplified from plasmid Δ*akuB*::*luc_OPT_*__red__*ptrA*_pUC19 (Seldeslachts et al., 2021; Resendiz-Sharpe et al., 2022) using oligonucleotides 9 and 10. The *nmt*1 sequence including 927 bp of its promoter and 260 bp of its terminator was amplified form gDNA of Af293 using oligonucleotides 11 and 12. By digesting Δ*akuB*::*luc_OPT_*__red__*ptrA*_pUC19 with *Not*I, the Δ*akuB*_pUC19 plasmid fragment was obtained and used for assembly with the gel-purified P*gpdAAn*:*luc_OPT_*__red_ *:*T*gpdAAn* and the *nmt1* sequence by *in vitro* recombination using the In-Fusion HD cloning kit (Takara/Clontech). The assembled plasmid was amplified in *E. coli* DH5α cells and reisolated. The plasmid was digested with *Sma*I and the Δ*akuB*::*luc_OPT_*__red__*nmt1* fragment was gel-purified and used for protoplast transformation of strain NIH4215. Transformants were regenerated on thiamine-free GG10 medium containing 1.2 M sorbitol and 0.4 mM of d-luciferin in the top-agar to select for thiamine prototrophic and bioluminescent transformants. To narrow down the candidates containing a deletion of the *akuB* gene, 60 transformants were screened by positional PCR for the absence of the *akuB* coding sequence and presence of the *nmt*1 sequence in the *akuB* locus by using oligonucleotides 15 and 16 or 15 and 17, respectively. A total of 10 mutants with the expected profile in the PCR analysis were further tested by Southern blot analysis. The gDNA from the selected transformants and the parental strain was digested with *BamH*I, separated on a 0.8% agarose gel and transferred to a nylon Hybond N+ membrane (GE Healthcare). A digoxygenin-labelled probe against the downstream region of the *akuB* gene was synthesised with oligonucleotides 18 and 19 using digoxygenin-labelled dUTPs (Roche) and Taq DNA polymerase (New England Biolabs). After hybridisation, the probe was detected by chemiluminescence imaging using anti-Digoxigenin-AP Fab-fragments for probe detection and CDP-Star as chemiluminescence substrate (Roche).

### Deletion of the *niaD* gene in bioluminescent reporter strains

For deletion of the *niaD* gene, a 943 bp upstream and a 953 bp downstream region of the *niaD* gene was amplified by PCR using oligonucleotides 25-28 and assembled by *in vitro* recombination in a *Sma*I digested pUC19 plasmid. The assembled Δ*niaD*_pUC19 plasmid was amplified in *E. coli* and positive clones were detected by colony PCR using oligonucleotides 28 and 29. The *ptrA* gene was excised from plasmid *ptrA*_pJet (Fleck and Brock, 2010), gel purified and ligated with the *Not*I restricted and dephosphorylated (FastAP; Thermo Scientific) plasmid Δ*niaD*_pUC19 using the Rapid DNA Ligation Kit (Thermo Scientific). Plasmids were amplified in *E. coli* and checked for correct assembly by colony PCR using oligonucleotides 29 and 30. The plasmid was digested with *Sma*I and the Δ*niaD::ptrA* construct was gel-eluted and used in the PEG-mediated transformation of protoplasts from Δ*akuB* strains 2/9 and 4/11. Transformants were selected by addition of 0.1 µg/ml pyrithiamine and 0.4 mM of d-luciferin to the transformation agar. Transformants were checked for homologous single-copy integration of the deletion construct by Southern blot analysis as described above. gDNA was digested with *Xho*I and a probe against the upstream region of *niaD* that was prepared using oligonucleotides 25 and 26.

### Generation of *pyrG* deletion mutants

For the deletion of the *pyr*G gene a 760 bp up- and a 770 bp downstream region of the *pyrG* gene were amplified from gDNA of NIH4215 using oligonucleotides 20-23. PCR products were gel-eluted and assembled with a *Sma*I-digested pUC19 plasmid by *in vitro* recombination. The resulting Δ*pyrG*_pUC19 plasmid was propagated in *E. coli* and the correct assembly was checked by colony PCR using the oligonucleotides 23 and 24. The *Not*I-restricted *ptrA* gene was ligated into the *Not*I-restricted and dephosphorylated Δ*pyrG*_pUC19 plasmid and the correct assembly was checked by colony PCR using oligonucleotides 20 and 30. The Δ*pyrG::ptrA*_pUC19 plasmid was restricted with *SmaI* and the gel-purified Δ*pyrG::ptrA* cassette was used for protoplast transformation of the Δ*akuB* strains 2/9 and 4/11. Transformants were regenerated on osmotically stabilised transformation medium supplemented with 10 mM uracil, 10 mM uridine and in selected plates in the presence of 2mg/ml of FOA. Southern blot analysis was performed as described above and gDNA of the parental strain and transformants was digested with *Hind*III. A probe against the upstream region of the *pyrG* gene was generated using oligonucleotides 20 and 21.

### Isolation and characterisation of spontaneous fluoroorotic acid mutants

Freshly harvested conidia suspensions of selected strains were adjusted to 1 × 10^8^ conidia per ml and 100 µl were plated on HEPES-buffered (20 mM, pH 7.0) *Aspergillus* minimal medium with glutamine as nitrogen sources and supplemented with 12.5 µg/ml of thiamine 4 mg/ml FOA. For each independent approach, five plates per strain were inoculated in parallel and plates were incubated for 7 days at 37°C. Conidia of resulting colonies were picked and transferred to fresh FOA-containing plates and incubated for 50 h prior to harvesting conidia for subsequent analyses. Randomly selected colonies were cultivated in liquid malt extract medium supplemented with thiamine and 10 mM uracil and the resulting mycelium was used for DNA extraction. Oligonucleotides 31 and 34 were used for amplification of the coding region of the *pyrG* gene and oligonucleotides 35 and 37 for amplification of the *pyrE* coding region. Phusion polymerase (Thermo) and high-fidelity buffer were used during the amplification. Amplified genes were gel-purified and sequenced by Eurofins with oligonucleotides 32 and 33 for the *pyrG* gene and 36 for the *pyrE* gene. Genomic DNA of the parental NIH4215 strain and strain CBS144.89 served as controls for gene amplification and sequencing. All sequences were aligned with the reference sequence of strain Af293 for the identification of potential gene mutations.

### Virulence studies in larvae of the greater wax moth *Galleria mellonella*

Conidia suspensions of strain CBS144.89 (Dal), its bioluminescent *KU^80^* deletion strain (*ΔakuB* N5) (Resendiz-Sharpe et al., 2022), as well as strain NIH4215 and its bioluminescent and *nmt1-*complemented *ΔakuB* strains 2/9 and 4/11 were freshly prepared from glucose/glutamine minimal medium slopes supplemented with or without thiamine. Conidia were harvested in PBS (Gibco) containing 0.01% Tween 20, washed twice and resuspended in PBS. Larvae of the greater wax moth *Galleria mellonella* (Waxworms Ltd - Sheffield, UK) were kept at 15°C for up to one week before starting the infection studies. To compare the virulence of wild-type strains and bioluminescent *ΔakuB* transformants, groups of 10 larvae weighing 200-300 mg were inoculated each with 50 µl PBS without (mock control) or with 1 × 10^5^, 2.5 × 10^5^, 5 × 10^5^ or 1 × 10^6^ conidia. Injections into the haemolymph were performed through the second pro-leg of *G. mellonella* larvae, using 0.5 mL BD Micro-Fine™ insulin syringes. In addition, a group of non-injected larvae served as treatment control. After infection the larvae were kept at 37°C in the dark and their survival was monitored daily for up to 6 days post-infection. The data was plotted in Kaplan-Meier survival curves and statistical analysis was performed by log-rank test using GraphPad Prism version 8.0.0 for Windows.

## Results

### Identification of thiamine auxotrophy of strain NIH4215

**Figure 1:**
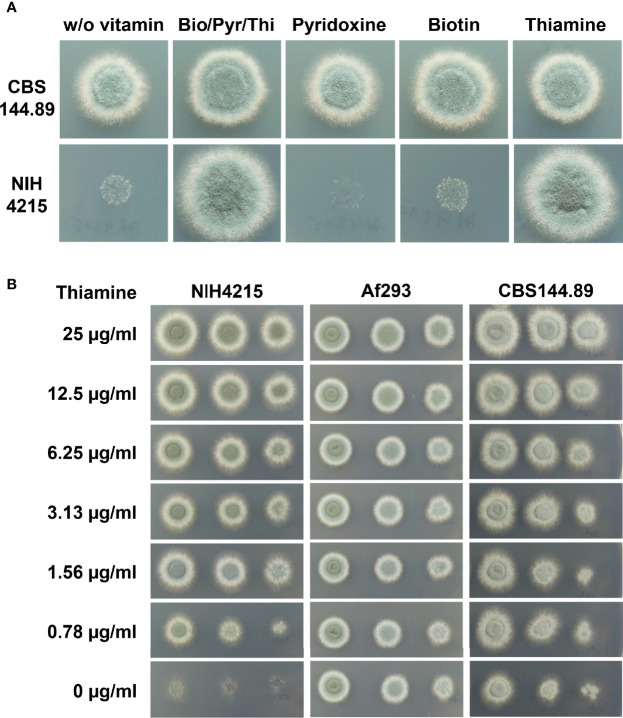
Growth of *A. fumigatus* strains on synthetic *Aspergillus* minimal media with or without vitamin supplementation. **(A)** 1 × 10^4^ conidia of strains CBS144.89 and NIH4215 were spotted and pictures were taken after 48 h of incubation at 37°C. Bio/Pyr/Thi indicates the supplementation with the three vitamins biotin, pyridoxin and thiamine. Strain NIH4215 strictly depends on the presence of thiamine for growth. **(B)** Media supplementation with a serial thiamine dilution. Strain NIH4215, Af293 and CBS144.89 were tested in parallel and 10-fold serial dilutions of conidia suspensions were spotted.

Strain NIH4215 was selected for generation of a bioluminescent reporter strain to increase the number of independent *A. fumigatus* strains available for *in vivo* drug efficacy studies using longitudinal imaging approaches (Resendiz-Sharpe et al., 2022). Previous studies on *A. fumigatus* NIH4215 generally used complex media such as Saboraud medium, cell-culture media or potato dextrose for strain maintenance, conidia production and antifungal drug testing (Petraitis et al., 2003; Hope et al., 2017). However, in *A. fumigatus* transformations, the use of synthetic minimal media is favoured as it allows the use of selection markers such as pyrithiamine (Kubodera et al., 2000). Unexpectedly, when spotting conidia of strain NIH4215 on synthetic minimal media, control strains such as the *A. fumigatus* Dal strain (CBS144.89) rapidly formed colonies, whereas only a very faint background growth was observed for NIH4215 (**Figure 1A**). To get insights on the supplements required for growth of this strain, we cultivated NIH4215 on various different media available in the laboratory, which included a synthetic minimal medium used for cultivation of *C. albicans*. This medium contained a vitamin supplementation of biotin, thiamine, and pyridoxine (Otzen et al., 2013). Surprisingly, NIH4215 grew on this medium and growth was also observed when this vitamin stock solution was used to supplement *Aspergillus* minimal medium (**Figure 1A**). Subsequent testing of the three individual vitamins revealed that thiamine supplementation was essential to restore growth of NIH4215 (**Figure 1A**). Therefore, this wild-type strain can be classified as a thiamine auxotrophic mutant. A supplementation with 0.78 µg/ml (about 2 µM) thiamine was the lowest concentration tested and showed that it was sufficient to support growth of NIH4215 (**Figure 1B**). Although growth at this low concentration was markedly reduced compared to higher concentrations, thiamine may have a growth promoting effect especially at the onset of germination as also strains Af293 and CBS144.89 germinated and grew slightly faster at elevated thiamine concentrations (**Figure 1B**).

### Identification of a putative thiamine biosynthesis pathway in *A. fumigatus*

**Figure 2:**
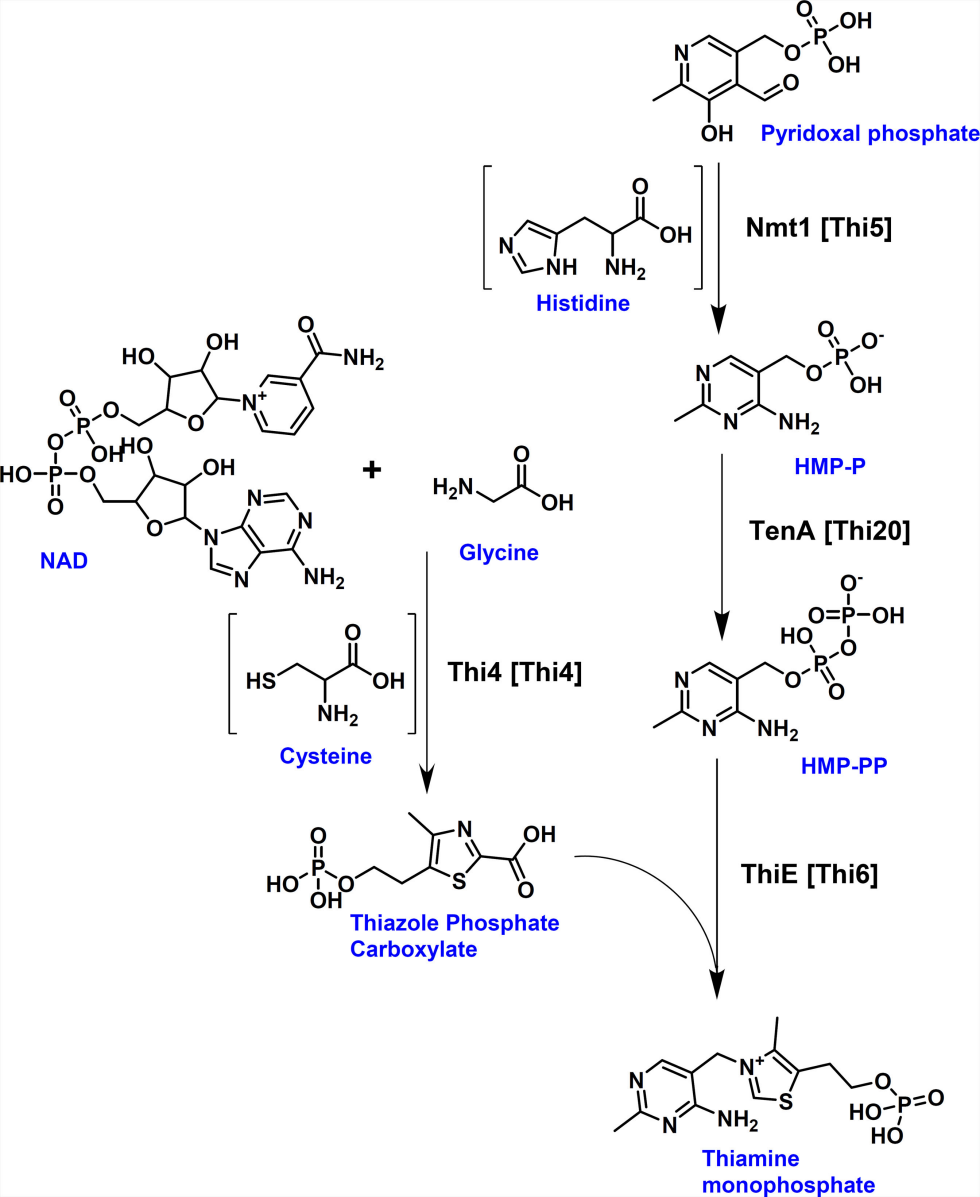
Fungal thiamine biosynthesis pathway. *Aspergillus* protein names are provided with corresponding yeast names given in brackets. [Thi4] = Thi4 = Thiamine thiazole synthase; [Thi5] = Nmt1 = 4-amino-5-hydroxymethyl-2-methylpyrimidine phosphate synthase; [Thi20] = TenA = phosphomethylpyrimidine kinase; [Thi6] = ThiE = hydroxyethyl thiazole kinase/thiamine-phosphate diphosphorylase. The pyrimidine and thiazole moieties are formed by two separate branches. Thi4 forms the thiazole moiety, whereby it uses the ADP-pentose moiety from NAD in combination with glycine and the sulphur group of an essential cysteine residue of Thi4 for the formation of the intermediate adenosine-diphospho-5-(β-ethyl)-4-methylthiazole-2-carboxylic acid (not shown), from which the thiazole phosphate carboxylate is released. This results in the inactivation of Thi4 due to the loss of the sulphur group from its cysteine residue (Chatterjee et al., 2011). Nmt1 (Thi5) catalyses the initial step in the formation of the pyrimidine moiety from pyridoxal phosphate and histidine resulting in the production of 4-amino-5-hydroxymethyl-2-methylpyrimidine phosphate (HMP-P) which is phosphorylated into its pyrophosphate (HMP-PP) by TenA (Thi20). ThiE (Thi6) joins the thiazole and the pyrimidine moieties under the formation of thiamine monophosphate.

Thiamine biosynthesis has been well studied in several yeasts such as *Saccharomyces cerevisiae* (Coquille et al., 2012) or *Candida albicans* (Lai et al., 2012). From these studies, it has been shown that thiamine biosynthesis derives from two branches. The pyrimidine moiety results from pyridoxal phosphate and an enzyme-derived histidine residue, whereas the thiazole moiety in fungi is formed from nicotinamide adenine dinucleotide, glycine and an enzyme-derived cysteine (**Figure 2**), whereby the respective thiamine azole synthase Thi4 becomes inactivated due to the incorporation of the sulphur group from the cysteine residue into the thiazole moiety (Chatterjee et al., 2011). By using the enzymes from the thiamine biosynthesis pathway of *S. cerevisiae* as templates, the protein database of *A. fumigatus* Af293 was searched for homologous proteins. The Nmt1 protein from *A. fumigatus* (accession: XP_748095; locus tag AFUA_5G02740) showed 67% identity to the 4-amino-5-hydroxymethyl-2-methylpyrimidine phosphate synthase Thi5 from *S. cerevisiae*. Use of the phosphomethylpyrimidine kinase Thi20 as template resulted in the identification of TenA (accession: XP_755470; locus tag AFUA_2G10740) from *A. fumigatus*. However, the sequence identity was only 33%, but the overall sequence similarity was at 50%. Furthermore, when using the bifunctional hydroxyethyl thiazole kinase/thiamine-phosphate diphosphorylase Thi6 as template a putative ThiE protein (accession: XP_755230; locus tag AFUA2G08970) with 35% sequence identity and 53% similarity was retrieved. Finally, using the thiamine thiazole synthase Thi4 as template a putative Thi4 protein (accession: XP_750752; locus tag AFUA_6G08360) with 60% sequence identity was identified in *A. fumigatus*. While experimental evidence for the contribution of these enzymes in *A. fumigatus* thiamine biosynthesis was not yet available, the identification of the putative Nmt1, TenA, ThiE and Thi4 proteins appeared sufficient for the biosynthesis of thiamine monophosphate from the respective precursor molecules (**Figure 2**).

### Genetic basis of thiamine auxotrophy in strain NIH4215

**Figure 3:**
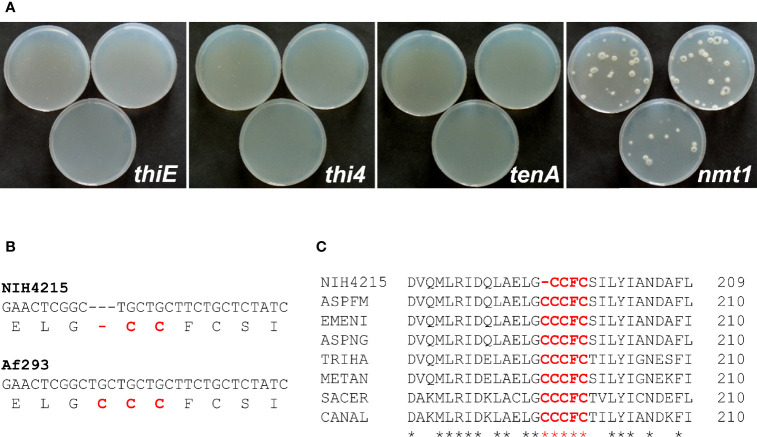
Identification of the molecular basis of thiamine auxotrophy of strain NIH4215. **(A)** Transformation of NIH4215 with thiamine biosynthesis genes from wild-type strain Af293. Pictures were taken at 72 h after transformation. Only the transformation with the *nmt1* gene restores thiamine prototrophy. **(B)** Partial DNA sequence alignment of *nmt1* genes from strains NIH4215 and Af293. The corresponding amino acid sequence is shown in 1-letter code. A cysteine codon present in the Af293 sequence is missing from the *nmt1* gene from strain NIH4215. **(C)** Partial protein sequence alignment of Nmt1 homologous from various fungal species. The iron-binding motif CCCFC (highlighted) is highly conserved with one cysteine residue lacking in the sequence of NIH4215. NIH4215, *A. fumigatus* NIH4215 Nmt1; ASPFM, *A. fumigatus* Af293 Nmt1 (accession: XP_748095); EMENI, *Aspergillus nidulans* Nmt1 (accession: XP_681278); ASPNG, *Aspergillus niger* Nmt1 (accession: XP_001400160); TRIHA, *Trichoderma harzianum* Thi11 (accession: KKP02383); METAN, *Metarhizium anisopliae* Nmt1 (accession: KFG80226); SACER, *S. cerevisiae* Thi5 (accession: AJU38966); CANAL, *Candida albicans* Thi5 (accession: C4YMW2).

With the identification of a set of genes potentially contributing to thiamine biosynthesis in *A. fumigatus*, we aimed to determine whether one of these genes was responsible for the observed thiamine auxotrophy of strain NIH4215. Thus, the respective genes including about 1 kb of their upstream and 200- 300 bp of downstream regions were amplified by PCR from genomic DNA of *A. fumigatus* Af293 and directly used for protoplasts transformation of strain NIH4215. Protoplasts were regenerated on medium without thiamine supplementation. While no colonies were obtained for transformations using the *tenA*, *thiE* or *thi4* genes, several colonies became visible on transformation plates in which the *nmt1* gene was used (**Figure 3A**). This indicated that all thiamine biosynthesis genes except that coding for Nmt1 are still functional in NIH4215. To determine whether a mutation in the coding region of *nmt1* was responsible for thiamine auxotrophy, the gene was amplified from genomic DNA of NIH4215 and sequenced. Surprisingly, sequence comparison with the gene from Af293 identified the lack of one out of three consecutive cysteine encoding TGC codons (**Figures 3B**, **C**). These cysteine residues are highly conserved in fungal THI5/Nmt1 proteins as they form part of a CCCFC iron-binding motif that is essential for enzymatic activity (Coquille et al., 2012). Since all other amino acids were identical to the Nmt1 sequence from Af293, the loss of this TGC codon and the accompanied loss of iron coordination in the mutated Nmt1 is the likely sole cause of thiamine auxotrophy in NIH4215.

### Generation of a bioluminescent *akuB* deletion mutant

**Figure 4:**
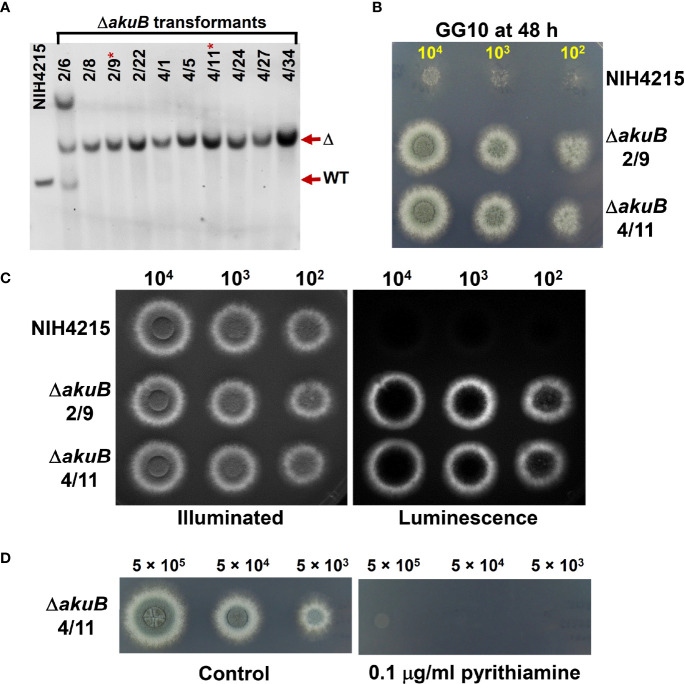
Southern blot and bioluminescence of Δ*akuB* strains generated by use of a wild-type *nmt1* gene as transformation marker. **(A)** Southern blot analysis of selected transformants. A digoxygenin-labelled probe against the downstream region of the *akuB* gene was used and genomic DNA was restricted by *Bam*HI resulting in expected signals at 2872 bp for the wild type (WT) and 4111 bp for deletion mutants (Δ). All transformants selected from PCR pre-screening showed the expected deletion signal with transformant 2/6 showing two additional integrations. Strains 2/9 and 4/11 (indicated by *) were selected for subsequent experiments. **(B)** Growth of the selected Δ*akuB* strains on thiamine-free minimal medium containing 50 mM glucose as carbon and 10 mM glutamine as nitrogen source (GG10). Serial dilutions of spore suspensions were spotted as indicated and pictures were taken after 48 h at 37°C. The *akuB* deletion mutants are prototrophic for thiamine. **(C)** Same as in **(B)**, but plates contained 25 µg/ml thiamine to enable growth of strain NIH4215 and 0.3 mM d-luciferin to allow for bioluminescence imaging. Left side picture is an acquisition under illumination and right side the luminescence acquisition in the dark. Note that pigmented conidia on top of colonies quench the light emission. Both selected strains show the expected bioluminescence, which is not observed for the parental strain. Pictures were taken after 42 h. **(D)** Test of pyrithiamine resistance of the selected Δ*akuB* strain 4/11. Conidia suspensions were spotted on GG10 medium with or without pyrithiamine supplementation. Introduction of the *nmt1* wild-type gene confers pyrithiamine sensitivity.

Since complementation of thiamine auxotrophy of strain NIH4215 was achieved by transformation with the *nmt1* gene from the wild-type strain Af293, we assumed that *nmt1* could act as a suitable marker for gene deletions in strain NIH4215. Therefore, we aimed in using the *nmt1* wild-type gene for the generation of a bioluminescent reporter strain by simultaneously replacing the coding region of the *akuB* locus in *A. fumigatus*. For that purpose, a thermostable red-shifted and codon-optimised version of the firefly luciferase (accession number: MT554554) under transcriptional control of the *A. nidulans gpdA* promoter and terminator was selected as previously used for generation of similar reporter strains in CBS144.89 (Resendiz-Sharpe et al., 2022). Downstream of the *gpdA* terminator the *nmt1* gene with its own promoter and terminator sequence was cloned and the entire cassette was flanked by the upstream and downstream regions of the *akuB* gene. This dual deletion/reporter cassette was excised from the plasmid backbone and used for transformation of *A. fumigatus* NIH4215. d-Luciferin in transformation plates allowed the visualisation and selection of bioluminescent strains and 60 transformants were analysed by positional PCR to identify candidates with an *akuB* locus deletion that was confirmed by Southern blot analysis of the pre-selected transformants (**Figure 4A**). Selected mutants were tested for thiamine prototrophy in comparison to the wild-type NIH4215 (**Figure 4B**) and showed colony formation on thiamine-free medium. Furthermore, the selected strains were tested for bioluminescence by spotting conidia suspension from selected Δ*akuB* strains and from the parental strain on medium containing thiamine and d-luciferin. While the parental strain NIH4215 is non-bioluminescent, the mycelium of transformants showed a bright signal, whereby areas covered with pigmented conidia remained dark due to dormancy of conidia and light absorption by melanin (**Figure 4C**). These experiment confirmed that the *nmt1* gene can be successfully used in gene deletion approaches and the approach resulted in “marker-free” bioluminescent *A. fumigatus* Δ*akuB* strains from strain NIH4215.

### Pyrithiamine sensitivity of *nmt1*-complemented NIH4215 strains

Pyrithiamine is toxic to several aspergilli and other ascomycetes when grown on thiamine-free media (Kubodera et al., 2002) as it suppresses the production of the enzyme cofactor thiamine pyrophosphate on several levels (Kubodera et al., 2000). In filamentous fungi, a major target of pyrithiamine is the thiazole synthase Thi4 and a pyrithiamine resistance-conferring *ptrA* gene that carries a point mutation in the *thi4* promoter region had previously been isolated from *Aspergillus oryzae* (Kubodera et al., 2000). While pyrithiamine is an excellent selection marker for *A. fumigatus* wild-type strains (Fleck and Brock, 2010), the marker was initially not available for use in the NIH4215 strain due to its need for thiamine supplementation. However, we speculated that complementation of the thiamine auxotrophy by integrating a wild-type *nmt1* gene as used in the generation of the bioluminescent Δ*akuB* strain might restore the ability to use pyrithiamine and, thus, the *ptrA* gene as selection marker. To test this hypothesis, we spotted different conidia concentrations of a selected Δ*akuB* strain on media with and without the addition of 0.1 µg/ml pyrithiamine (**Figure 4D**). As expected, the strain was sensitivity towards pyrithiamine. This indicated that spending the *nmt1* marker for generation of the bioluminescent Δ*akuB* strain freed the *ptrA* gene as new selection marker.

### Exploitation of the *ptrA* gene for production of *pyrG* and *niaD* deletion mutants in the bioluminescent reporter strain

**Figure 5:**
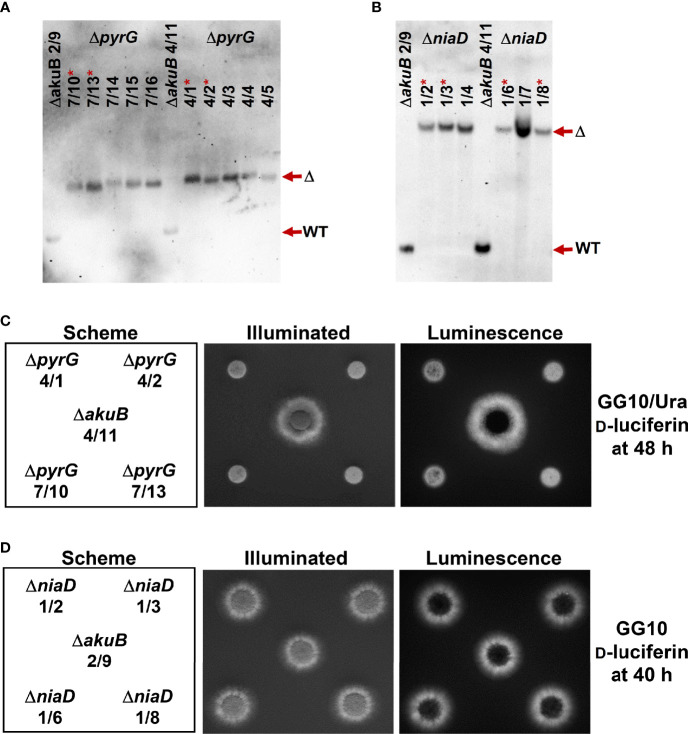
Southern blot and bioluminescence analysis of Δ*pyrG* and Δ*niaD* mutants generated from bioluminescent Δ*akuB* strains. **(A, B)** Southern blot analyses of randomly selected transformants obtained from the parental Δ*akuB* strains 2/9 and 4/11. The parental strains provide show the regions of the respective wild-type signals (WT) of *pyrG* and *niaD*. **(A)** Δ*pyrG* detection with a digoxygenin-labelled probe against the upstream region of the *pyrG* gene. Genomic DNA was restricted with *Hind*III resulting in expected signals of 2239 bp for the wild-type (WT) and 4056 bp for deletion mutants (Δ). **(B)** Δ*niaD* detection with a digoxygenin-labelled probe against the upstream region of the *niaD* gene. Genomic DNA was restricted with *Xho*I resulting in signals expected at 1635 bp for the wild-type (WT) and 6258 bp for deletion mutants (Δ). Strains in **(A)** and **(B)** denoted by a * were analysed in subsequent experiments. **(C)** Growth of the selected Δ*pyrG* strains on minimal medium containing 50 mM glucose as carbon and 10 mM glutamine as nitrogen source (GG10) and supplemented with 10 mM uracil and 0.3 mM d-luciferin. 10^4^ conidia were spotted and plates were incubated for 48 h at 37°C before luminescence was detected. All Δ*pyrG* strains show reduced growth rates, but are positive for bioluminescence. **(D)** Bioluminescence analysis of Δ*niaD* strains. Plates were the same as in **(C)**, but without uracil supplementation. On this medium, no growth defect of Δ*niaD* is detected and all strains are positive for bioluminescence. In **(C, D)** a spotting scheme is shown on the left side with one parental Δ*akuB* strain in the centre. The picture in the middle shows an acquisition under illumination and the right side a luminescence acquisition in the dark.

**Figure 6:**
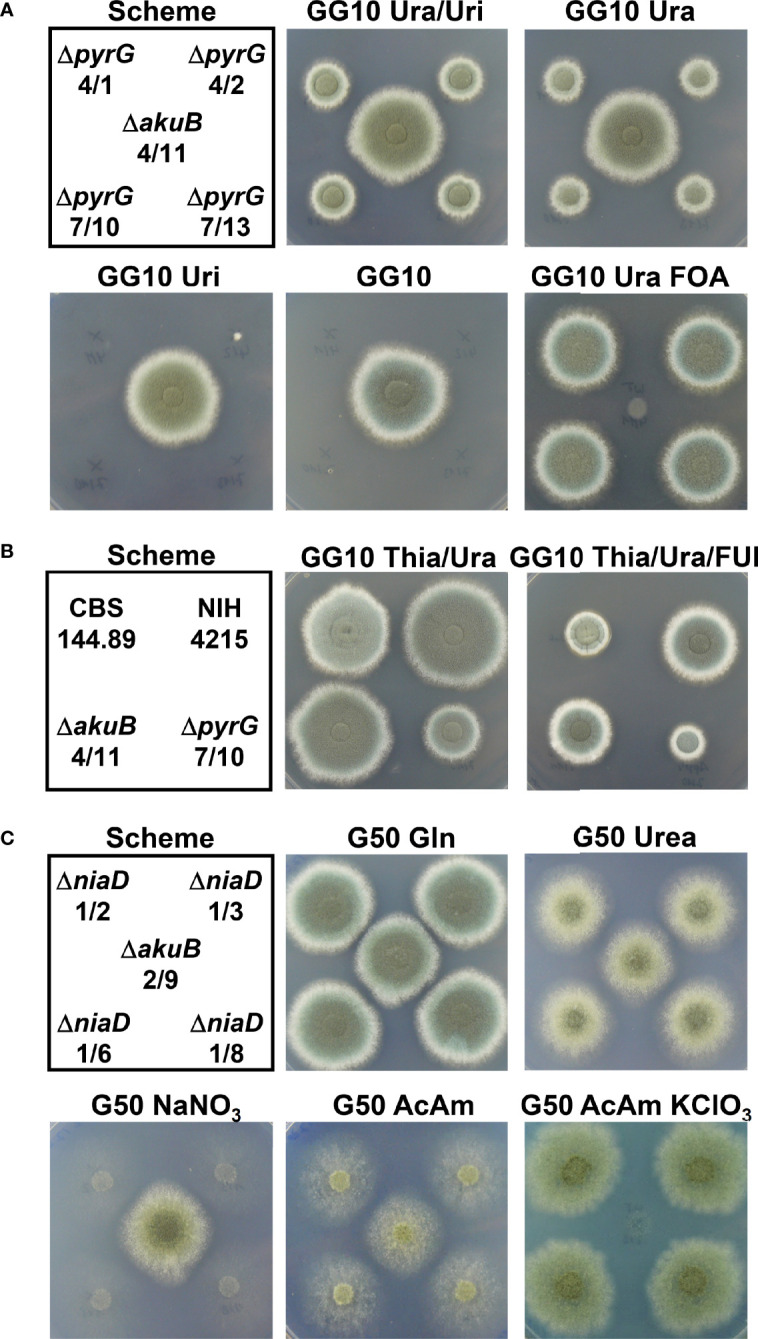
Growth analysis of Δ*pyrG* and Δ*niaD* mutants derived from the bioluminescent Δ*akuB* strains. **(A)** Growth analysis of Δ*pyrG* strains in comparison to the parental Δ*akuB* 4/11 strain. A spotting scheme for 10^4^ conidia from each strain is shown on the left. Glucose minimal medium with 10 mM glutamine as nitrogen source (GG10) served as basic growth medium. Media were additionally supplemented with either a combination of uracil and uridine (Ura/Uri, 10 mM each) or uracil (Ura, 10 mM) or uridine (Uri, 10 mM) alone. In addition, resistance against 4 mg/ml FOA in the presence of uracil was tested. All pictures were taken after 60 h of incubation at 37°C except for the FOA-containing plate that was photographed after 78 h. Uridine does not complement the Δ*pyrG* phenotype and growth in the presence or uracil is delayed. All *pyrG* mutants show the expected resistance against FOA. **(B)** Test of the *A. fumigatus* wild-type strain CBS144.89, the NIH4215 strain and the derived Δ*akuB* 4/11 and the Δ*pyrG* 7/10 strain against 5-fluorouridine (FUI). GG10 medium supplemented with thiamine and uracil served as control and was compared at 64 h against a medium additionally supplemented with 2 mM FUI. Although growth of all strains is affected by FUI, strain NIH4215 and derivatives show normal colony morphology and appear less affected. **(C)** Growth analysis of selected Δ*niaD* strains in comparison to the parental Δ*akuB* 2/9 strain. A spotting scheme for 10^4^ conidia from each strain is shown on the left. Glucose minimal medium (G50) served as basis and was supplemented with different nitrogen sources. Gln, 10 mM glutamine; Urea, 10 mM urea; NaNO_3_, 70 mM sodium nitrate; AcAm, 20 mM acetamide; AcAm KClO_3_, 20 mM acetamide + 120 mM potassium chlorate. All pictures were taken after 60 h of incubation at 37°C except for the chlorate –containing plate that was photographed after 120 h. All *niaD* mutants show the expected inability to utilise nitrate, but show an increased chlorate resistance.

The *pyrG* and *niaD* genes are perfect markers for fungal transformation as they allow a marker recycling by mitotic out-recombination followed by a positive selection process. *pyrG* (or *URA3* in *S. cerevisiae*) encodes the orotidine-5’-monophosphate decarboxylase that also converts 5-fluoroorotic acid (FOA) monophosphate into the toxic metabolite 5-fluoroorotidine monophosphate (Boeke et al., 1984). Therefore, while *pyrG* negative strains require the supplementation of media with uracil and/or uridine, they are resistant against FOA (D’enfert et al., 1996). Flanking of a *pyrG* cassette by direct DNA repeats allows a mitotic out-recombination event that results in the loss of the *pyrG* cassette and leads back to uracil/uiridine auxotrophic strains. FOA selection can then be used to screen for the respective strains (Geib et al., 2019). While *pyrG* negative *A. fumigatus* strains are non-pathogenic, re-introduction of the *pyrG* gene restores virulence (D’enfert et al., 1996). The *niaD* gene encodes a nitrate reductase and is essential for the use of nitrate as nitrogen source (Unkles et al., 1992). Similar to the *pyrG* gene, a positive selection of mutants with defect in the *niaD* gene is possible by their ability to grow in the presence of chlorate (Ishi et al., 2005). Thus, we generated deletion constructs for the *pyrG* (accession XP_755170) and *niaD* (XP_752653.1) gene from *A. fumigatus* using the pyrithiamine resistance gene *ptrA* as selection marker. Both deletion constructs were used for the transformation of two independent bioluminescent Δ*akuB* strains. Individual transformants were picked and tested by Southern blot analysis, which showed that all strains contained the desired gene deletion (**Figures 5A**, **B**). Subsequently, selected strains were analysed for bioluminescence (**Figures 5C**, **D**) and growth phenotypes under a variety of conditions (**Figure 6**). While the *pyrG* mutants showed the expected requirement of uracil, growth was slower than that of the parental strain. Most strikingly, uridine, given as sole supplement, did not restore growth and a mixture of uracil and uridine did not increase the growth speed of the mutants (**Figure 6A**). This was surprising since *Aspergillus niger* and *Aspergillus oryzae pyrG* mutants can be complemented by uridine (Geib et al., 2019) and the *pyrG*-negative *A. fumigatus* mutants CEA17 and CEA22 derived from strain CBS144.89 have also been described to use uridine in the complementation of the *pyrG* negative phenotype (D’enfert, 1996; D’enfert et al., 1996). Despite the reduced growth rate of the *pyrG* mutants on uracil supplemented media, all strains were positive for bioluminescence (**Figure 5C**). In addition, the mutants formed colonies on uracil-supplemented media in the presence of FOA, confirming the suitability of the *pyrG* marker in positive selection approaches. To further investigate the inability to use uridine, we speculated that strain NIH4215 might possess an increased resistance towards 5-fluorouridine (FUI) when compared to the wild-type strain CBS144.89. The addition of 2 mM of FUI retarded growth of all strains (**Figure 6B**), but the growth reduction was more pronounced for strain CBS144.89 compared to NIH4215 and its derived mutants. This indicates at least some level of increased FUI resistance of NIH4215 in line with its inability to utilise uridine.

All Δ*niaD* strains grew well on media with glutamine as nitrogen source and showed a similar bioluminescence as the parental strain (**Figure 5C**). As expected, the *niaD* mutants were unable to use nitrate as nitrogen source (**Figure 6C**). However, normal growth was observed on the nitrogen sources glutamine and urea, and growth on the less preferred nitrogen source acetamide was unsuspicious when compared to the parental strain (**Figure 6B**). Growth on acetamide was analysed as it appeared as a suitable nitrogen source when testing the chlorate sensitivity in positive selection approaches since preferred nitrogen sources such as ammonium or glutamine cause an AreA-dependent repression of the nitrate reductase (Amaar and Moore, 1998). Indeed, chlorate repressed growth of the parental strain, but allowed colony formation and sporulation of the *niaD* mutants (**Figure 6C**). In summary, *pyrG* and *niaD* mutants were generated with high frequency in the Δ*akuB* strain and the mutants showed the expected auxotrophy for either uracil or nitrate. The introduced markers can be used for positive selection and all strains remained bioluminescent, allowing their use in *in vivo* imaging approaches.

### Virulence analysis in a *Galleria mellonella* infection model

**Figure 7:**
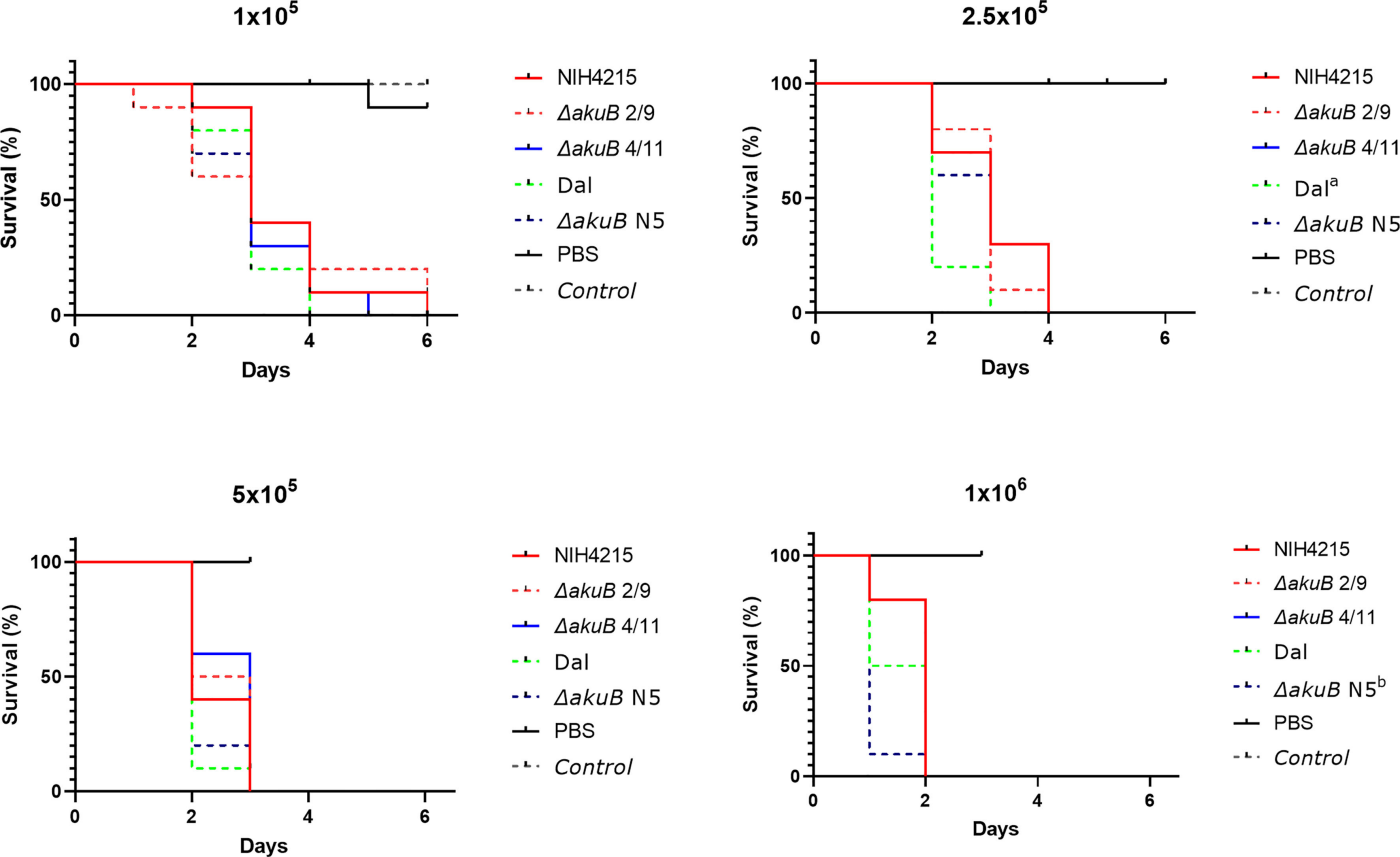
Virulence analysis of NIH4215 and *nmt1*-complemented bioluminescent Δ*akuB* strains. Kaplan-Meier survival curves of *Galleria mellonella* larvae infected with *A. fumigatus* wild-type strains NIH4215 and CBS144.89 (Dal) and the corresponding bioluminescent *ΔakuB* mutants (*ΔakuB* N5 for Dal; *ΔakuB* 2/9 and *ΔakuB* 4/11 for NIH4215). Each larva was injected with 50 µl of PBS or 50 µl PBS containing either 1 × 10^5^, 2.5 × 10^5^, 5 × 10^5^ or 1 × 10^6^ conidia of the individual strains. A group of untreated larvae was included as general viability control (Control). Significant differences in survival rates was tested by log-rank analysis. The NIH4215 wild type and its *ΔakuB* 2/9 and *ΔakuB* 4/11 strains did not significantly differ in their killing rate over the range of conidia concentrations tested. In the survival curve at 2.5 × 10^5^ conidia/larva the Dal strain showed a significant difference compared to the NIH4215 strain indicated by ^a^ (*p* = 0.0149). At 2.5 × 10^5^ conidia/larva the *ΔakuB* N5 strain differed significantly to the Dal strain (*p* = 0.0331) and at 1 × 10^6^ conidia/larva to strain NIH4215 (*p* = 0.0022) as indicated by ^b^.

Due to the unexpected thiamine auxotrophy of strain NIH4215, we were interested in the virulence of the auxotrophic strain in comparison to its *nmt1*-complemented bioluminescent Δ*akuB* reporter strains. As most simple virulence model we selected larvae of the greater wax moth *Galleria mellonella* for pathogenicity studies (Durieux et al., 2021). Since *A. fumigatus* strain CBS144.89 and its corresponding bioluminescent Δ*akuB* reporter had already been shown to possess identical virulence potential in murine infection models (Resendiz-Sharpe et al., 2022), these strains were added as virulence controls. Four different concentrations of conidia in the range between 1 × 10^5^ and 1 × 10^6^ were used for infection and larvae were incubated for a maximum of 6 days at which even at the lowest concentration a mortality of up to 100% was reached. As expected (**Figure 7**), a concentration dependent killing of larvae was observed with all larvae succumbing to infection at day 2 when infected with 1 × 10^6^ conidia, at day 3 with 5 × 10^5^ conidia, at day 4 with 2.5 × 10^5^ conidia and between days 4 and 6 with 1 × 10^5^ conidia. No significant differences in survival rates among all strains was observed at the lowest and most representative dose of 1 × 10^5^ conidia. In one group of the higher concentrations the CBS144.89 (Dal) strain and at another concentration its Δ*akuB* strain showed a statistical difference in survival rates to NIH4215 or to NIH4215 and Dal strain, respectively, but due to the short time from infection to death of all larvae these differences appear less relevant. Most importantly, the thiamine auxotrophic NIH4215 strain showed no attenuated virulence in this infection model and the *akuB* deletion combined with the expression of the bioluminescent reporter had no negative impact. This indicates that the host supplies sufficient thiamine for germination and growth of a thiamine auxotrophic mutant.

### Spontaneous phenotypic mutations in NIH4215 and its derivatives

**Figure 8:**
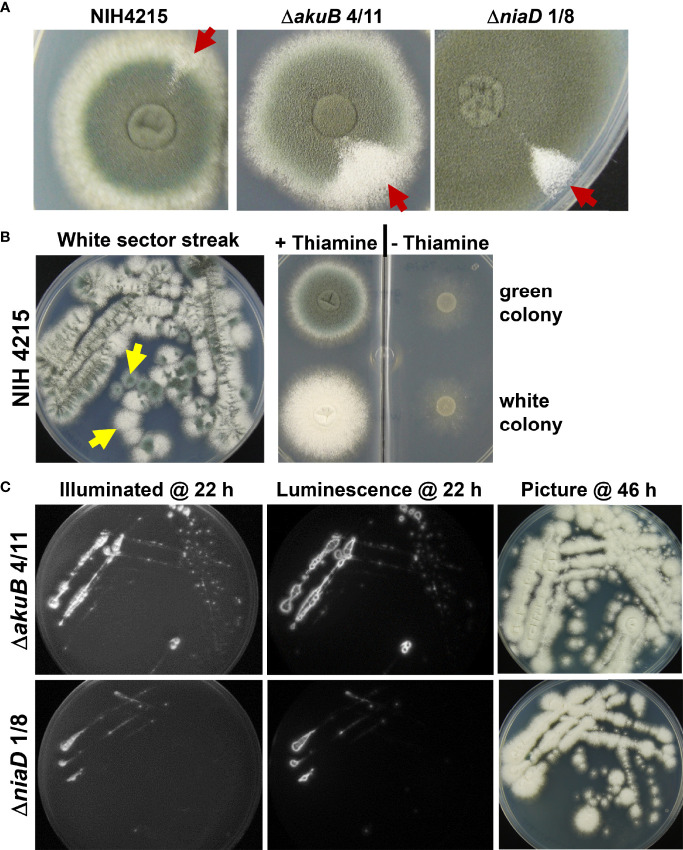
Phenotypic analysis of strains deriving from spontaneously occurring white colony sectors. **(A)** Visualisation of white sectors (arrows) arising on the edges of colonies of the parental strain NIH2415 and different transformants. Spores from the sectors were collected for further analysis. **(B)** Streak from white sector of the NIH2415 colony on thiamine containing glucose minimal medium at 46 h. Due to the small sector, a mixture of green and white colonies are growing. Spores from a green and a white single colony (arrows) were transferred to a petri dish with two compartments, one with and one without thiamine containing medium. At 48 h, the green and the white colony both show the expected thiamine auxotrophy of the parental strain. **(C)** Analysis of bioluminescence and conidia colouration of white sectors streaks from the Δ*akuB* and the Δ*niaD* strain. Conidia from sectors were transferred to glucose minimal medium containing 0.3 mM d-luciferin and were incubated at 37°C. Luminescence was analysed after 22 h at which initial mycelium formation becomes visible. The mature colonies with white conidia are shown after 46 h of incubation.

While thiamine auxotrophy of strain NIH4215 showed no impact on virulence, further observations indicated that this isolate seems to be prone for increased mutation rates. Besides thiamine auxotrophy and an inability to utilise uridine, morphological changes in colony sectors such as a more condensed colony morphology, fluffy sectors with increased aerial hyphae or sectors without conidia formation were observed (not shown). However, the most obvious phenotype was the spontaneous appearance of white colony sectors, which is indicative for mutations in the DHN-melanin biosynthesis pathway (Brakhage and Liebmann, 2005). This was observed for the NIH4215 wild-type strain as well as for the bioluminescent Δ*akuB* strains and mutants thereof (**Figure 8A**). To confirm that these sectors represent a stable mutation in the DHN-melanin pigment biosynthesis pathway, we picked conidia from white sectors of the parental NIH4215 strain, an Δ*akuB* strain and from a derived Δ*niaD* mutant and streaked them on fresh media either supplemented with thiamine (for NIH4215) or without thiamine, but with d-luciferin (for Δ*akuB* and Δ*niaD*). Due to the small white sector of the NIH4215 strain at the time of picking, the streak showed a mixture of green and white colonies (**Figure 8B**), but when individual colonies were picked and tested for growth in the absence of thiamine, green and white colonies were both auxotrophic for thiamine (**Figure 8B**). For the Δ*akuB* and Δ*niaD* strain, plates from streaks were imaged after 22 h and showed a strong bioluminescence signal for both white sectors streaks (**Figure 8C**). This analysis confirmed the origin of the white mutants from their parental bioluminescent reporter strains. When photographed after 46 h at which time conidia had formed on top of the colonies, the resulting colonies where white (**Figure 8C**). As white sectors spontaneously occurred in the parental NIH4215 strain and in the bioluminescent strains generated in this study, these mutations were not dependent on the deletion of the *akuB* locus and pointed towards a high rate of spontaneous mutations in this clinical isolate.

### Comparative analysis of NIH4215 mutation rates

**Figure 9:**
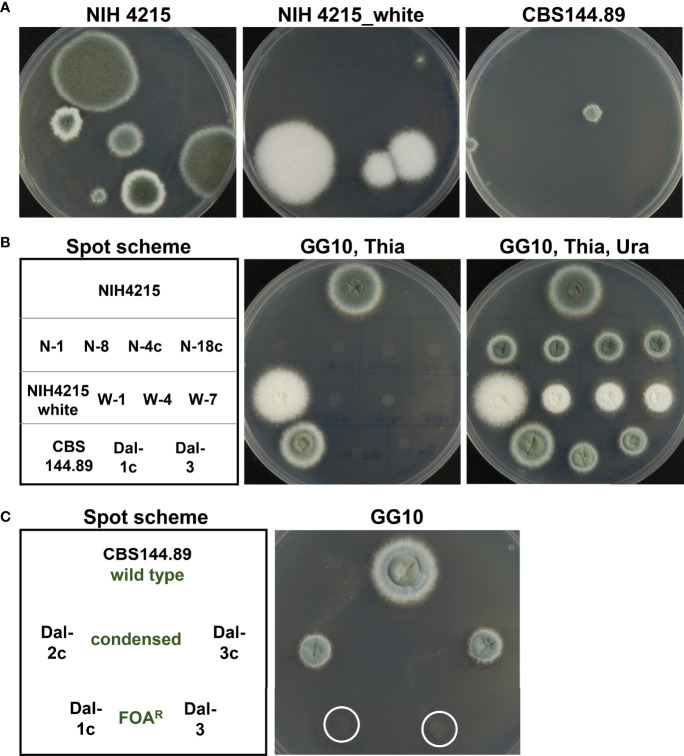
Screen for fluoroorotic acid resistant mutants. **(A)** Five HEPES-buffered (20 mM, pH 7.0) plates containing GG10 medium supplemented with 12.5 µg/ml thiamine, 10 mM uracil and 4 mg/ml fluoroorotic acid (FOA) were inoculated with 2 × 10^7^ conidia of strain NIH4215, the white NIH4215 mutant, or strain CBS144.89 and incubated for 7 days at 37°C. Between 2 and 6 FOA resistant colonies per plate were obtained for NIH4215 and its white mutant and between 0 and 3 colonies per plate with a condensed phenotype for CBS144.89. Representative photographs of plates are shown. **(B)** Analysis of selected FOA-resistant strains for uracil auxotrophy. For CBS144.89 non-condensed growing FOA-resistant mutants from various screens (see main text) were selected. For genotypes of FOA-resistant strains from NIH4215 and CBS144.89 strains refer to **Table 2**. **(C)** Analysis of Uracil auxotrophy of colonies from CBS144.89 with condensed and non-condensed phenotype. The mutants with condensed phenotype do not require uracil, but maintain the condensed colony morphology on minimal media without FOA.

While the repeated isolation of white mutants from NIH4215 and its derivatives indicated a generally high mutation rate, this phenotype was difficult to quantify, as there was no positive selection method available to determine the frequency of this mutation. Since we were able to show that positive FOA selection worked well in the *pyrG* deletion strains (**Figure 6A**), we exploited the FOA resistance for determination of spontaneously occurring mutations. However, it is worth to note that in aspergilli two genes are required to convert fluoroorotic acid into the toxic metabolite fluorouridine monophosphate as previously shown for *Aspergillus oryzae* (Mao et al., 2015). In a first step, the orotate phosphoribosyltransferase PyrE converts fluoroorotic acid into fluoroorotidine monophosphate, which is then decarboxylated by the orotidine monophosphate decarboxylase PyrG into fluorouridine monophosphate (Mao et al., 2015). Thus, a loss-of-function mutation in either the *pyrE* or *pyrG* gene leads to uracil auxotrophy accompanied by FOA resistance. By contrast, an albino phenotype only requires a mutation in a single gene coding for the polyketide synthase PksP - also called Alb1 (Watanabe et al., 2000; Gibbons et al., 2022). However, an albino mutation may occur at much higher frequency than the phenotype of FOA resistance from the combined mutation rates from *pyrE* and *pyrG*. The *pksP*/*alb1* gDNA spans 6662 bp and codes for a 2146 amino acid protein (accession Q4WZA8). By contrast, the *pyrE* gene has a size of 807 bp, contains a 66 bp intron and codes for a 246 amino acid protein (accession: XP_755462.1). The *pyrG* gene spans 905 bp, contains a 68 bp intron and codes for a protein of 278 amino acids (accession XP_755170.1). This means that the *pksP* gene encodes about 4 times more amino acids than the *pyrE* and *pyrG* gene combined. In addition, with a genome size of 29.4 Mbp (Nierman et al., 2005) the combined *pyrE* and *pyrG* gene size of 1712 bp only occupies a small fraction of the genome at which mutations may have an effect on FOA resistance. As only mutations that cause a frame shift (base insertion or base deletion) or that lead to a loss-of-function mutation will cause FOA resistance, we expected that even with an increased mutation rate the recovery of FOA-resistant mutants might be low. Indeed, in a first approach, we inoculated five FOA plates each with 1 × 10^6^ conidia, but no FOA resistant mutants were obtained. In a second approach, we inoculated five plates with 2 × 10^7^ conidia each (total of 1 × 10^8^ conidia) and obtained a total of 11 FOA resistant mutants for strain NIH4215. When we repeated the experiment with the same conidia suspension, 13 FOA resistant mutants were recovered, showing that the result from this conidia suspension was reproducible. We then prepared a fresh and independent conidia suspension and this time 18 FOA resistant mutants were retrieved (**Figure 9**). Similarly, when we prepared a conidia suspension from the white NIH4215 mutant we obtained 15 FOA resistant strains. To confirm that the FOA resistant mutants from strain NIH4215 were not clonal rather than resulting from independent mutation events, we randomly selected mutants for sequencing of the *pyrE* and *pyrG* gene (**Table 2**). Indeed, while a few duplicates were found, a broad spectrum of different mutations (transitions, transversions, base insertions, base deletions) were observed. When we plated the same amount of conidia from strain CBS144.89, we observed none or some condensed growing colonies that, when tested further, did not show a uracil/uridine auxotrophy, which indicates that these colonies did not carry a mutation in either *pyrE* or *pyrG* (**Figure 9**). When we plated this conidia suspension again, besides the condensed growing colonies a single FOA resistant mutant was retrieved that revealed a point mutation in the *pyrE* gene (**Table 2**). We then prepared a fresh conidia suspension of CBS144.89, but this time added uridine to the agar slopes. When this conidia suspension was tested, we obtained a total of 18 FOA resistant colonies, but when three colonies were randomly selected and sequenced, they all showed the identical mutation in the *pyrE* gene, which was a G to T transversion at the intron boarder of the *pyrE* gene. This transversion prevents intron splicing, resulting in a change of the amino acid sequence and introduces a stop codon (**Table 2**). This indicates that these mutants all derived from a single mutation event. Thus, while it is difficult to define an exact DNA mutation rate without next generation genome sequencing analyses (Zhu et al., 2014), our analyses indicate that in strain NIH4215 the likelihood to obtain independent FOA-resistant mutants is < 1 × 10^-7^, whereas this likelihood is > 1 × 10^-8^ in strain CBS144.89. This confirms that strain NIH4215 has a generally increased mutation rate compared to other *A. fumigatus* wild-type strains.

**Table 2:** Mutation analysis of fluoroorotic acid resistant mutants.

Strain	Clone	Gene	Mutation (gDNA)	Effect (Protein)
NIH4215	N-1	*pyrE*	A626G	E187G
NIH4215	N-7	*pyrG*	G652A	G195E
NIH4215	N-8	*pyrG*	ΔA653	Frame shift @ pos.195
NIH4215	N-9	*pyrE*	Δ465-480	Δ134-138 (ΔGEGGN)
NIH4215	N-3c	*pyrE*	C445A	R127S
NIH4215	N-4c	*pyrE*	C619A	R185S
NIH4215	N-5c	*pyrE*	C619A	R185S
NIH4215	N-7c	*pyrE*	C445T	R127C
NIH4215	N-17c	*pyrE*	A insertion @ pos. 32	Frameshift @ pos. 10
NIH4215	N-18c	*pyrG*	G246T	V60F
CBS144.89	Dal-1^a^	*pyrE*	G270T	Intron splicing
CBS144.89	Dal-3^a^	*pyrE*	G270T	Intron splicing
CBS144.89	Dal-8 ^a^	*pyrE*	G270T	Intron splicing
CBS144.89	Dal-1c	*pyrE*	T insertion @ pos. 219	Frameshift @ pos. 72

a Conidia suspension derived from uridine-containing slope.

## Discussion

*A. fumigatus* strain NIH4215 has frequently been used as wild-type strain in drug efficacy studies. Therefore, we were surprised to see that the strain was unable to grow on synthetic minimal medium and required the addition of thiamine. Previous studies did not discover this auxotrophy since strain NIH4215 grows without obvious phenotype on complex thiamine containing media such as potato dextrose broth, Sabouraud medium, Malt extract or cell culture medium. Thiamine, also called vitamin B1, is an essential cofactor in many catabolic reactions and a deficiency in humans causes cardiovascular diseases, dementia and other severe neurological conditions (Gibson et al., 2016; Chandrakumar et al., 2018; Dinicolantonio et al., 2018). By contrast, plants and most microorganisms including fungi are able to synthesise thiamine *de novo* as it plays an essential role in the pyruvate dehydrogenase and the α-ketoglutarate dehydrogenase complex, is vital for the degradation of branched-chain amino acids and forms part of the transketolase in the pentose phosphate pathway (Bettendorff and Wins, 2013). It also plays an essential role in the pyruvate decarboxylase (Robinson and Chun, 1993), which is required in yeasts for ethanol production during fermentative growth. Due to this central role of thiamine in primary metabolism, it could have been assumed that the virulence of strain NIH4215 is reduced. However, at least in our insect infection model, no attenuation of virulence was detected, which implies that the host provides sufficient thiamine to promote germination and growth of a thiamine biosynthesis deficient pathogen. In addition, we observed that strain NIH4215 seems to have an efficient thiamine uptake system since less than 1 µg/ml of thiamine still supported growth albeit the growth rate was slightly reduced when compared to high thiamine supplementation levels. Increased thiamine concentrations have been shown to cause a beneficial effect on stress tolerance in yeasts (Wolak et al., 2014; Wolak et al., 2015) and the same may apply to *A. fumigatus*. Both thiamine prototrophic *A. fumigatus* wild-type strains, Af293 and CBS144.89, showed increased germination and growth rate in the presence of elevated thiamine concentrations. The faster germination could also indicate a limitation of the suicide enzyme Thi4 from the thiazole biosynthesis branch (Chatterjee et al., 2011) in germinating conidia, which is compensated by thiamine supplementation.

While thiamine auxotrophy has not previously been described for a clinical *A. fumigatus* isolate, varying levels of thiamine auxotrophy have been described for several pathogenic yeasts and dimorphic fungi. Growth of *C. albicans*, *C. dubliniensis*, *C. tropicalis* and *C. glabrata* is significantly impaired in thiamine-free minimal media, whereby the strongest thiamine dependency was observed for *C. glabrata* (Wolak et al., 2015). Similarly, while *Histoplasma capsulatum*, a fungal pathogen that can replicate within the phagosome of macrophages, depends on functional biosynthesis pathways for pantothenate and ribovlavin, it does not require thiamine biosynthesis for intraphagosomal replication (Garfoot et al., 2014). Accordingly, studies on the nutritional requirements of this dimorphic pathogen revealed that different levels of thiamine auxotrophy were widespread among a collection of 25 *H. capsulatum* isolates with some isolates deriving from human infections (Mcveigh and Morton, 1965). Thiamine auxotrophy has also been described for several *Paracoccidioides brasiliensis* isolates (San Blas and Centeno, 1977) which is the major cause of Paracoccidiomycosis in Latin America (Ferreira, 2009). This shows that thiamine requirement is frequently observed in pathogenic yeasts and dimorphic fungi but has not been described for wild-type isolates of a saprophytic and generally free-living filamentous fungus. However, the lack of thiamine requirement during infection implies that the host environment provides sufficient thiamine to complement the auxotrophy and is in agreement with our results from *G. mellonella* infections. This further indicates that thiamine biosynthesis is unlikely to provide a new and suitable antifungal drug target. Nevertheless, for topical applications such as skin infections caused by *Malassezia* species the use of the antivitamin oxythiamine has been discussed as it significantly reduced the growth rate of *Malassezia pachydermatis* in complex growth media (Siemieniuk et al., 2016).

When searching for the molecular basis of thiamine auxotrophy in strain NIH4215, we identified the deletion of an entire cysteine codon in the *nmt1* gene of NIH4215. This deletion disrupts an unusual CCCFC iron-binding motif in the 4-amino-5-hydroxymethyl-2-methylpyrimidine phosphate synthase and studies on the related *S. cerevisiae* Thi5p enzyme showed that individual mutations of each of the four cysteine residues into alanine caused thiamine auxotrophy (Coquille et al., 2012). Thus, the deletion of the cysteine codon in the *nmt1* gene confirmed the importance of this iron-binding motif in the enzyme from a filamentous fungus. While this explained the observed thiamine auxotrophy of strain NIH4215, it also opened the opportunity to use a wild-type version of the *nmt1* gene as auxotrophic marker to generate a bioluminescent *A. fumigatus* reporter strain by simultaneously deleting the *akuB* locus for increased frequency of homologous recombination.

The firefly luciferase is a perfect tool to investigate fungal virulence, disease progression, dissemination of infection and therapy efficacy in murine infection models as it allows quantification of the fungal burden in temporal and spatial resolution *via* a non-invasive bioluminescence imaging (Slesiona et al., 2012; Jacobsen et al., 2014; Poelmans et al., 2018; Persyn et al., 2019; Vanherp et al., 2019; Vanherp et al., 2020; Seldeslachts et al., 2021). Specifically the use of a red-shifted version of the firefly luciferase increases sensitivity of fungal detection as it reduces the light absorption from haemoglobin in deep-seated infections (Resendiz-Sharpe et al., 2022). Furthermore, in combination with other non-invasive imaging techniques such as micron-scale computed tomography (micro-CT) and magnetic resonance imaging (MRI), bioluminescence imaging enables to correlate the fungal burden with the formation of tissue lesions and inflammation in infected organs (Van Dyck et al., 2020). While the *nmt1* gene served as excellent marker for transformation, the rate of homologous integration in the NIH4215 strain was only around 17%, which is in agreement with other *A. fumigatus* strains with an intact non-homologous end-joining repair mechanism (Krappmann et al., 2006). The resulting luciferase-expressing Δ*akuB* mutants displayed no obvious growth or morphological defects, showed no virulence attenuation in the *G. mellonella* infection model, were prototrophic for thiamine and showed the expected bioluminescence. Therefore, these strains can be used as “marker-free” bioluminescent reporters for further infection and gene deletion studies. Importantly, while the *nmt1* marker was no longer available for a second round of transformation, we were now able to exploit the pyrithiamine resistance gene as selection marker as this marker only works in strains with an intact thiamine biosynthesis pathway (Kubodera et al., 2000). As a proof-of-concept, we decided to delete the *pyrG* and *niaD* gene from two selected bioluminescent Δ*akuB* strains to show (i) that the pyrithiamine resistance gene can be used in the reporter strains and (ii) to confirm the high rate of homologous integration and (iii) to generate strains with auxotrophic markers that allow a positive selection in marker recycling steps. Indeed, all transformants analysed contained a single homologous integration into the respective locus. While deletion of the *niaD* gene resulted in the expected inability to use nitrate as nitrogen source and mediated an increased chlorate resistance, the *pyrG* mutant showed a strict dependency on uracil supplementation and was resistant to fluoroorotic acid.

Unexpectedly, the Δ*pyrG* strains were unable to use uridine for complementation of the *pyrG* mutation. Uridine is an uracil base that is attached to a ribose unit (Uracil-1-β-d-ribofuranoside) and therefore shows increased water solubility compared to uracil and is a generally a good metabolite for use in the complementation of *pyrG* negative *Aspergillus* strains (Geib et al., 2019). However, in pyrimidine biosynthesis the ribose unit of uridine needs either phosphorylation or removal for further metabolism. Since uracil, but not uridine supported growth of the *pyrG* mutant, it seems that the strain NIH4215 possesses a mutation that either affects the uptake of uridine, the phosphorylation of uridine and/or the deglycosylation of uridine. In *S. cerevisiae*, the uridine permease Fui1p (ORF YBL042) is responsible for uridine uptake and a deletion leads to increased resistance against 5-fluorouridine (Wagner et al., 1998). The subsequent phosphorylation of uridine in *S. cerevisiae* proceeds *via* the uridine kinase Urk1p (Kurtz et al., 2002). However, a mutation in Urk1p is not sufficient to cause a uridine-negative phenotype a simultaneous mutation in the uridine nucleosidase (Urh1p in *S. cerevisiae*) is required as this enzyme removes the ribose unit from uridine to produce uracil (Kurtz et al., 2002). We did not follow the precise cause for the lack of uridine utilisation in NIH4215 Δ*pyrG* mutants, but the strong resistance of NIH4215 and its derivatives against 5-fluorouridine indicates that the lack of uridine utilisation was already present in the parental clinical isolate.

Strain NIH4215 appears specifically prone to increased mutation rates and future studies may involve sequencing of the genome of this isolate to investigate potential mutations in the DNA replication proofreading machinery and to search for further mutations that may have accumulated in its genome. In this respect, during prolonged growth of individual strains generated in this study, we observed the sudden appearance of white sectors, which indicates the spontaneous occurrence of mutations in the DHN-melanin biosynthesis pathway (Perez-Cuesta et al., 2020) and is in agreement with previously observed frameshift mutations in clinical albino *A. fumigatus* isolates (Gibbons et al., 2022). To confirm an increased mutation rate in NIH4215, we aimed in the comparative analysis of mutation rates. While precise analysis of mutation rates, including silent mutations, requires whole genome sequencing (Zhu et al., 2014), we exploited the FOA resistance caused by functional mutations in the *pyrE* and *pyrG* gene (Mao et al., 2015). As a result, we found that compared to strain CBS144.89 the isolation of FOA resistant strains from NIH4215 was increased by at least a factor of ten. Besides nucleotide transversions and transitions in the *pyrG* and *pyrE* gene resulting in loss-of-function mutations, we also detected single nucleotide insertions and deletions and in mutant N-9 we detected a 15 bp deletion in the *pyrE* gene resulting in a five amino acids deletion from PyrE. Such a deletion event of more than a single nucleotide is in line with the deletion of an entire cysteine codon in the *nmt1* gene. Due to this obviously high mutation rate, we wondered whether the thiamine auxotrophy in NIH4215 occurred during the early activation of our stock culture. Therefore, we tested an independent NIH4215 cryostock (kindly provided by Thomas Lehrnbecher, Goethe University Frankfurt/Main, Germany) that was also auxotrophic for thiamine (not shown). This confirmed that the thiamine dependency was not a special feature of the sample analysed in this study.

We conclude from our analyses that the thiamine auxotrophy of strain NIH4215 does not negatively influence the results from previous studies performed with this strain. Thiamine biosynthesis appears dispensable during host infection and additional mutations will only accumulate when the strain is repeatedly subcultivated. However, the detection of an increased mutation rate of NIH4215 makes the wild-type strain and the derived reporter strains less attractive for future virulence studies. Nevertheless, NIH4215 and the reporter strains may be valuable tools to investigate the spontaneous occurrence of azole resistance. Azole resistance in clinical *A. fumigatus* isolates is a growing problem (Meis et al., 2016) and it has been assumed that most mutations causing this resistance accumulate in the environment (Meis et al., 2016; Resendiz Sharpe et al., 2018). Azole resistant strains have been found on all continents except Antarctica (Burks et al., 2021), but it is unclear whether the most frequently observed resistance-conferring mutations derived from only a few progenitors or developed *de novo* in individual strains. If the latter is the case, a mutation-prone strain such as NIH4215 may be an ideal candidate to study the development of azole resistance *in vitro* and *in vivo* by a similar approach as used for the selection of FOA resistant mutants.

In summary, thiamine biosynthesis does not appear to be of special importance for pathogenic fungi to establish infection in insects or humans, as thiamine auxotrophy is common in pathogenic yeasts and present in the clinical *A. fumigatus* isolate investigated in this study. A number of different clinical *A. fumigatus* isolates are in use for infection studies, but our analysis shows that individual isolates may have accumulated not only obvious, but also cryptic mutations that may affect results. While there is a great value in the use of clinical isolates from different sources, strains should be carefully analysed for growth defects on minimal media and care should be taken when drawing conclusions on the contribution of virulence determinants and efficacy of antifungal drugs when results base on a single and not well-characterised clinical isolate.

## Data availability statement

The original contributions presented in the study are included in the article/supplementary material. Further inquiries can be directed to the corresponding author.

## Author contributions

RPS and MB designed and performed all experiments and evaluated the data. MB drafted the manuscript and both authors agreed to the content of the final manuscript version.

## Funding

This work was financially supported by the Medical Research Council (MRC) grant MR/N017528/1 to MB and RPS.

## Conflict of interest

The authors declare that the research was conducted in the absence of any commercial or financial relationships that could be construed as a potential conflict of interest.

## Publisher’s note

All claims expressed in this article are solely those of the authors and do not necessarily represent those of their affiliated organizations, or those of the publisher, the editors and the reviewers. Any product that may be evaluated in this article, or claim that may be made by its manufacturer, is not guaranteed or endorsed by the publisher.

## References

[B1] Altschul S. F. Gish W. Miller W. Myers E. W. Lipman D. J. (1990). Basic local alignment search tool. J. Mol. Biol. 215, 403–410. doi: 10.1016/S0022-2836(05)80360-2 2231712

[B2] Amaar Y. G. Moore M. M. (1998). Mapping of the nitrate-assimilation gene cluster (*crnA-niiA-niaD*) and characterization of the nitrite reductase gene (*niiA*) in the opportunistic fungal pathogen *Aspergillus fumigatus* . Curr. Genet. 33, 206–215. doi: 10.1007/s002940050328 9508795

[B3] Bettendorff L. Wins P. (2013). “Biochemistry of thiamine and thiamine phosphate compounds,” in Encyclopedia of biological chemistry, 2nd ed. Eds. Lennarz W. J. Lane M. D. (Academic Press), 202–209.

[B4] Boeke J. D. Lacroute F. Fink G. R. (1984). A positive selection for mutants lacking orotidine-5’-phosphate decarboxylase activity in yeast: 5-fluoro-orotic acid resistance. Mol. Gen. Genet. 197, 345–346. doi: 10.1007/BF00330984 6394957

[B5] Box H. Livermore J. Johnson A. Mcentee L. Felton T. W. Whalley S.. (2016). Pharmacodynamics of isavuconazole in a dynamic *In vitro* model of invasive pulmonary aspergillosis. Antimicrob. Agents Chemother. 60, 278–287. doi: 10.1128/AAC.01364-15 26503648 PMC4704219

[B6] Brakhage A. A. Liebmann B. (2005). *Aspergillus fumigatus* conidial pigment and cAMP signal transduction: significance for virulence. Med. Mycol. 43 Suppl 1, S75–S82. doi: 10.1080/13693780400028967 16110796

[B7] Brock M. Jouvion G. Droin-Bergere S. Dussurget O. Nicola M. A. Ibrahim-Granet O. (2008). Bioluminescent *Aspergillus fumigatus*, a new tool for drug efficiency testing and *in vivo* monitoring of invasive aspergillosis. Appl. Environ. Microbiol. 74, 7023–7035. doi: 10.1128/AEM.01288-08 18820063 PMC2583481

[B8] Burks C. Darby A. Gomez Londono L. Momany M. Brewer M. T. (2021). Azole-resistant *Aspergillus fumigatus* in the environment: Identifying key reservoirs and hotspots of antifungal resistance. PloS Pathog. 17, e1009711. doi: 10.1371/journal.ppat.1009711 34324607 PMC8321103

[B9] Chandrakumar A. Bhardwaj A. T Jong G. W. (2018). Review of thiamine deficiency disorders: Wernicke encephalopathy and korsakoff psychosis. J. Basic Clin. Physiol. Pharmacol. 30, 153–162. doi: 10.1515/jbcpp-2018-0075 30281514

[B10] Chang P. K. (2008). A highly efficient gene-targeting system for *Aspergillus parasiticus* . Lett. Appl. Microbiol. 46, 587–592. doi: 10.1111/j.1472-765X.2008.02345.x 18346134

[B11] Chatterjee A. Abeydeera N. D. Bale S. Pai P. J. Dorrestein P. C. Russell D. H.. (2011). *Saccharomyces cerevisiae* THI4p is a suicide thiamine thiazole synthase. Nature 478, 542–546. doi: 10.1038/nature10503 22031445 PMC3205460

[B12] Coquille S. Roux C. Fitzpatrick T. B. Thore S. (2012). The last piece in the vitamin B1 biosynthesis puzzle: structural and functional insight into yeast 4-amino-5-hydroxymethyl-2-methylpyrimidine phosphate (HMP-p) synthase. J. Biol. Chem. 287, 42333–42343. doi: 10.1074/jbc.M112.397240 23048037 PMC3516776

[B13] Da Silva Ferreira M. E. Kress M. R. Savoldi M. Goldman M. H. Hartl A. Heinekamp T.. (2006). The *akuB*(KU80) mutant deficient for nonhomologous end joining is a powerful tool for analyzing pathogenicity in *Aspergillus fumigatus* . Eukaryot. Cell 5, 207–211. doi: 10.1128/EC.5.1.207-211.2006 16400184 PMC1360264

[B14] Dellaporta S. L. Wood J. Hicks J. B. (1983). A plant DNA minipreparation: Version II. Plant Mol. Biol. Rep. 1, 19–21. doi: 10.1007/BF02712670

[B15] D’enfert C. (1996). Selection of multiple disruption events in *Aspergillus fumigatus* using the orotidine-5’-decarboxylase gene, *pyrG*, as a unique transformation marker. Curr. Genet. 30, 76–82. doi: 10.1007/s002940050103 8662213

[B16] D’enfert C. Diaquin M. Delit A. Wuscher N. Debeaupuis J. P. Huerre M.. (1996). Attenuated virulence of uridine-uracil auxotrophs of *Aspergillus fumigatus* . Infect. Immun. 64, 4401–4405. doi: 10.1128/iai.64.10.4401-4405.1996 8926121 PMC174389

[B17] Dinicolantonio J. J. Liu J. O’keefe J. H. (2018). Thiamine and cardiovascular disease: A literature review. Prog. Cardiovasc. Dis. 61, 27–32. doi: 10.1016/j.pcad.2018.01.009 29360523

[B18] Durieux M. F. Melloul E. Jemel S. Roisin L. Darde M. L. Guillot J.. (2021). *Galleria mellonella* as a screening tool to study virulence factors of *Aspergillus fumigatus* . Virulence 12, 818–834. doi: 10.1080/21505594.2021.1893945 33682618 PMC7946008

[B19] Ferreira M. S. (2009). Paracoccidioidomycosis. Paediatr. Respir. Rev. 10, 161–165. doi: 10.1016/j.prrv.2009.08.001 19879504

[B20] Fleck C. B. Brock M. (2010). *Aspergillus fumigatus* catalytic glucokinase and hexokinase: expression analysis and importance for germination, growth, and conidiation. Eukaryot. Cell 9, 1120–1135. doi: 10.1128/EC.00362-09 20453072 PMC2901669

[B21] Galiger C. Brock M. Jouvion G. Savers A. Parlato M. Ibrahim-Granet O. (2013). Assessment of efficacy of antifungals against *Aspergillus fumigatus*: value of real-time bioluminescence imaging. Antimicrob. Agents Chemother. 57, 3046–3059. doi: 10.1128/AAC.01660-12 23587947 PMC3697358

[B22] Garfoot A. L. Zemska O. Rappleye C. A. (2014). *Histoplasma capsulatum* depends on *de novo* vitamin biosynthesis for intraphagosomal proliferation. Infect. Immun. 82, 393–404. doi: 10.1128/IAI.00824-13 24191299 PMC3911860

[B23] Geib E. Baldeweg F. Doerfer M. Nett M. Brock M. (2019). Cross-chemistry leads to product diversity from atromentin synthetases in aspergilli from section *Nigri* . Cell Chem. Biol. 26, 223–234.e226. doi: 10.1016/j.chembiol.2018.10.021 30527997

[B24] Gibbons J. G. D’avino P. Zhao S. Cox G. W. Rinker D. C. Fortwendel J. R.. (2022). Comparative genomics reveals a single nucleotide deletion in *pksP* that results in white-spore phenotype in natural variants of *Aspergillus fumigatus* . Front. Fungal Biol. 3. doi: 10.3389/ffunb.2022.897954 PMC10512363 37746219

[B25] Gibson G. E. Hirsch J. A. Fonzetti P. Jordan B. D. Cirio R. T. Elder J. (2016). Vitamin B1 (thiamine) and dementia. Ann. N Y Acad. Sci. 1367, 21–30. doi: 10.1111/nyas.13031 26971083 PMC4846521

[B26] Hope W. W. Mcentee L. Livermore J. Whalley S. Johnson A. Farrington N.. (2017). Pharmacodynamics of the orotomides against *Aspergillus fumigatus*: New opportunities for treatment of multidrug-resistant fungal disease. mBio 8, e01157–17. doi: 10.1128/mBio.01157-17 28830945 PMC5565967

[B27] Ishi K. Watanabe T. Juvvadi P. R. Maruyama J. Kitamoto K. (2005). Development of a modified positive selection medium that allows to isolate *Aspergillus oryzae* strains cured of the integrated *niaD*-based plasmid. Biosci. Biotechnol. Biochem. 69, 2463–2465. doi: 10.1271/bbb.69.2463 16377911

[B28] Jacobsen I. D. Luttich A. Kurzai O. Hube B. Brock M. (2014). *In vivo* imaging of disseminated murine *Candida albicans* infection reveals unexpected host sites of fungal persistence during antifungal therapy. J. Antimicrob. Chemother. 69, 2785–2796. doi: 10.1093/jac/dku198 24951534

[B29] Kousha M. Tadi R. Soubani A. O. (2011). Pulmonary aspergillosis: a clinical review. Eur. Respir. Rev. 20, 156–174. doi: 10.1183/09059180.00001011 21881144 PMC9584108

[B30] Krappmann S. Sasse C. Braus G. H. (2006). Gene targeting in *Aspergillus fumigatus* by homologous recombination is facilitated in a nonhomologous end- joining-deficient genetic background. Eukaryot. Cell 5, 212–215. doi: 10.1128/EC.5.1.212-215.2006 16400185 PMC1360265

[B31] Kubodera T. Yamashita N. Nishimura A. (2000). Pyrithiamine resistance gene (*ptrA*) of *Aspergillus oryzae*: cloning, characterization and application as a dominant selectable marker for transformation. Biosci. Biotechnol. Biochem. 64, 1416–1421. doi: 10.1271/bbb.64.1416 10945258

[B32] Kubodera T. Yamashita N. Nishimura A. (2002). Transformation of *Aspergillus* sp. and *Trichoderma reesei* using the pyrithiamine resistance gene (*ptrA*) of *Aspergillus oryzae* . Biosci. Biotechnol. Biochem. 66, 404–406. doi: 10.1271/bbb.66.404 11999416

[B33] Kurtz J. E. Exinger F. Erbs P. Jund R. (2002). The URH1 uridine ribohydrolase of *Saccharomyces cerevisiae* . Curr. Genet. 41, 132–141. doi: 10.1007/s00294-002-0296-9 12111094

[B34] Lai R. Y. Huang S. Fenwick M. K. Hazra A. Zhang Y. Rajashankar K.. (2012). Thiamin pyrimidine biosynthesis in *Candida albicans*: a remarkable reaction between histidine and pyridoxal phosphate. J. Am. Chem. Soc. 134, 9157–9159. doi: 10.1021/ja302474a 22568620 PMC3415583

[B35] Mao Y. Yin Y. Zhang L. Alias S. A. Gao B. Wei D. (2015). Development of a novel *Aspergillus* uracil deficient expression system and its application in expressing a cold-adapted α-amylase gene from Antarctic fungi *Geomyces pannorum* . Process Biochem. 50, 1581–1590. doi: 10.1016/j.procbio.2015.06.016

[B36] Mcveigh I. Morton K. (1965). Nutritional studies of *Histoplasma capsulatum* . Mycopathol. Mycol. Appl. 25, 294–308. doi: 10.1007/BF02049917 5877181

[B37] Meis J. F. Chowdhary A. Rhodes J. L. Fisher M. C. Verweij P. E. (2016). Clinical implications of globally emerging azole resistance in *Aspergillus fumigatus* . Philos. Trans. R Soc. Lond. B Biol. Sci. 371, 20150460. doi: 10.1098/rstb.2015.0460 28080986 PMC5095539

[B38] Montrucchio G. Lupia T. Lombardo D. Stroffolini G. Corcione S. De Rosa F. G.. (2021). Risk factors for invasive aspergillosis in ICU patients with COVID-19: current insights and new key elements. Ann. Intensive Care 11, 136. doi: 10.1186/s13613-021-00923-4 34524562 PMC8441237

[B39] Nierman W. C. Pain A. Anderson M. J. Wortman J. R. Kim H. S. Arroyo J.. (2005). Genomic sequence of the pathogenic and allergenic filamentous fungus *Aspergillus fumigatus* . Nature 438, 1151–1156. doi: 10.1038/nature04332 16372009

[B40] Otzen C. Müller S. Jacobsen I. D. Brock M. (2013). Phylogenetic and phenotypic characterisation of the 3-ketoacyl-CoA thiolase gene family from the opportunistic human pathogenic fungus *Candida albicans* . FEMS Yeast Res. 13, 553–564. doi: 10.1111/1567-1364.12057 23758791

[B41] Pagano L. Akova M. Dimopoulos G. Herbrecht R. Drgona L. Blijlevens N. (2011). Risk assessment and prognostic factors for mould-related diseases in immunocompromised patients. J. Antimicrob. Chemother. 66 Suppl 1, i5–14. doi: 10.1093/jac/dkq437 21177404

[B42] Perez-Cuesta U. Aparicio-Fernandez L. Guruceaga X. Martin-Souto L. Abad-Diaz-De-Cerio A. Antoran A.. (2020). Melanin and pyomelanin in *Aspergillus fumigatus*: from its genetics to host interaction. Int. Microbiol. 23, 55–63. doi: 10.1007/s10123-019-00078-0 31020477

[B43] Persyn A. Rogiers O. Brock M. Vande Velde G. Lamkanfi M. Jacobsen I. D.. (2019). Monitoring of fluconazole and caspofungin activity against *In vivo Candida glabrata* biofilms by bioluminescence imaging. Antimicrob. Agents Chemother. 63, e01555–18. doi: 10.1128/AAC.01555-18 30420485 PMC6355587

[B44] Petraitis V. Petraitiene R. Sarafandi A. A. Kelaher A. M. Lyman C. A. Casler H. E.. (2003). Combination therapy in treatment of experimental pulmonary aspergillosis: synergistic interaction between an antifungal triazole and an echinocandin. J. Infect. Dis. 187, 1834–1843. doi: 10.1086/375420 12792859

[B45] Poelmans J. Himmelreich U. Vanherp L. Zhai L. Hillen A. Holvoet B.. (2018). A multimodal imaging approach enables *In vivo* assessment of antifungal treatment in a mouse model of invasive pulmonary aspergillosis. Antimicrob. Agents Chemother. 62, e00240–18. doi: 10.1128/AAC.00240-18 29760132 PMC6021662

[B46] Resendiz-Sharpe A. Da Silva R. P. Geib E. Vanderbeke L. Seldeslachts L. Hupko C.. (2022). Longitudinal multimodal imaging-compatible mouse model of triazole-sensitive and -resistant invasive pulmonary aspergillosis. Dis. Model. Mech. 15. doi: 10.1242/dmm.049165 PMC8990085 35352801

[B47] Resendiz Sharpe A. Lagrou K. Meis J. F. Chowdhary A. Lockhart S. R. Verweij P. E.. (2018). Triazole resistance surveillance in *Aspergillus fumigatus* . Med. Mycol. 56, 83–92. doi: 10.1093/mmy/myx144 29538741 PMC11950814

[B48] Robinson B. H. Chun K. (1993). The relationships between transketolase, yeast pyruvate decarboxylase and pyruvate dehydrogenase of the pyruvate dehydrogenase complex. FEBS Lett. 328, 99–102. doi: 10.1016/0014-5793(93)80973-X 8344439

[B49] San Blas F. Centeno S. (1977). Isolation and preliminary characterization of auxotrophic and morphological mutants of the yeast-like form of *Paracoccidioides brasiliensis* . J. Bacteriol. 129, 138–144. doi: 10.1128/jb.129.1.138-144.1977 830638 PMC234906

[B50] Schrevens S. Sanglard D. (2021). Investigating *Candida glabrata* urinary tract infections (UTIs) in mice using bioluminescence imaging. J. Fungi (Basel) 7, 844. doi: 10.3390/jof7100844 34682265 PMC8538756

[B51] Seldeslachts L. Vanderbeke L. Fremau A. Resendiz-Sharpe A. Jacobs C. Laeveren B.. (2021). Early oseltamivir reduces risk for influenza-associated aspergillosis in a double-hit murine model. Virulence 12, 2493–2508. doi: 10.1080/21505594.2021.1974327 34546839 PMC8923074

[B52] Shi C. Shan Q. Xia J. Wang L. Wang L. Qiu L.. (2022). Incidence, risk factors and mortality of invasive pulmonary aspergillosis in patients with influenza: A systematic review and meta-analysis. Mycoses 65, 152–163. doi: 10.1111/myc.13410 34882852 PMC9306612

[B53] Siemieniuk M. Czyzewska U. Strumilo S. Tylicki A. (2016). Thiamine antivitamins–an opportunity of therapy of fungal infections caused by *Malassezia pachydermatis* and *Candida albicans* . Mycoses 59, 108–116. doi: 10.1111/myc.12441 26691773

[B54] Slesiona S. Ibrahim-Granet O. Olias P. Brock M. Jacobsen I. D. (2012). Murine infection models for *Aspergillus terreus* pulmonary aspergillosis reveal long-term persistence of conidia and liver degeneration. J. Infect. Dis. 205, 1268–1277. doi: 10.1093/infdis/jis193 22438397

[B55] Such K. A. Petraitis V. Petraitiene R. Strauss G. E. Moradi P. W. Walsh T. J. (2013). Environmental monitoring for *Aspergillus fumigatus* in association with an immunosuppressed rabbit model of pulmonary aspergillosis. J. Am. Assoc. Lab. Anim. Sci. 52, 541–544. 24041208 PMC3784658

[B56] Takahashi T. Masuda T. Koyama Y. (2006). Enhanced gene targeting frequency in *ku70* and *ku80* disruption mutants of *Aspergillus sojae* and *Aspergillus oryzae* . Mol. Genet. Genomics 275, 460–470. doi: 10.1007/s00438-006-0104-1 16470383

[B57] Unkles S. E. Campbell E. I. Punt P. J. Hawker K. L. Contreras R. Hawkins A. R.. (1992). The *Aspergillus niger niaD* gene encoding nitrate reductase: upstream nucleotide and amino acid sequence comparisons. Gene 111, 149–155. doi: 10.1016/0378-1119(92)90682-F 1541396

[B58] Vande Velde G. Kucharikova S. Van Dijck P. Himmelreich U. (2018). Bioluminescence imaging increases *in vivo* screening efficiency for antifungal activity against device-associated *Candida albicans* biofilms. Int. J. Antimicrob. Agents 52, 42–51. doi: 10.1016/j.ijantimicag.2018.03.007 29572043

[B59] Van Dyck K. Rogiers O. Vande Velde G. Van Dijck P. (2020). Let’s shine a light on fungal infections: A noninvasive imaging toolbox. PloS Pathog. 16, e1008257. doi: 10.1371/journal.ppat.1008257 32134998 PMC7058284

[B60] Vanherp L. Poelmans J. Hillen A. Janbon G. Brock M. Lagrou K.. (2020). The added value of longitudinal imaging for preclinical *In vivo* efficacy testing of therapeutic compounds against cerebral cryptococcosis. Antimicrob. Agents Chemother. 64. doi: 10.1128/AAC.00070-20 PMC7318040 32284382

[B61] Vanherp L. Ristani A. Poelmans J. Hillen A. Lagrou K. Janbon G.. (2019). Sensitive bioluminescence imaging of fungal dissemination to the brain in mouse models of cryptococcosis. Dis. Model. Mech. 12, e00070–20. doi: 10.1242/dmm.039123 PMC6602310 31101657

[B62] Wagner R. De Montigny J. De Wergifosse P. Souciet J. L. Potier S. (1998). The ORF YBL042 of *Saccharomyces cerevisiae* encodes a uridine permease. FEMS Microbiol. Lett. 159, 69–75. doi: 10.1111/j.1574-6968.1998.tb12843.x 9485596

[B63] Watanabe A. Fujii I. Tsai H. Chang Y. C. Kwon-Chung K. J. Ebizuka Y. (2000). *Aspergillus fumigatus alb1* encodes naphthopyrone synthase when expressed in *Aspergillus oryzae* . FEMS Microbiol. Lett. 192, 39–44. doi: 10.1111/j.1574-6968.2000.tb09356.x 11040426

[B64] Wolak N. Kowalska E. Kozik A. Rapala-Kozik M. (2014). Thiamine increases the resistance of baker’s yeast *Saccharomyces cerevisiae* against oxidative, osmotic and thermal stress, through mechanisms partly independent of thiamine diphosphate-bound enzymes. FEMS Yeast Res. 14, 1249–1262. doi: 10.1111/1567-1364.12218 25331172

[B65] Wolak N. Tomasi M. Kozik A. Rapala-Kozik M. (2015). Characterization of thiamine uptake and utilization in *Candida* spp. subjected to oxidative stress. Acta Biochim. Pol. 62, 445–455. doi: 10.18388/abp.2015_1044 26284264

[B66] Zhang J. Mao Z. Xue W. Li Y. Tang G. Wang A.. (2011). *Ku80* gene is related to non-homologous end-joining and genome stability in *Aspergillus niger* . Curr. Microbiol. 62, 1342–1346. doi: 10.1007/s00284-010-9853-5 21225265

[B67] Zhang C. Meng X. Wei X. Lu L. (2016). Highly efficient CRISPR mutagenesis by microhomology-mediated end joining in *Aspergillus fumigatus* . Fungal Genet. Biol. 86, 47–57. doi: 10.1016/j.fgb.2015.12.007 26701308

[B68] Zhu Y. O. Siegal M. L. Hall D. W. Petrov D. A. (2014). Precise estimates of mutation rate and spectrum in yeast. Proc. Natl. Acad. Sci. U.S.A. 111, , E2310–2318. doi: 10.1073/pnas.1323011111 24847077 PMC4050626

